# Formulation, Stability, Pharmacokinetic, and Modeling Studies for Tests of Synergistic Combinations of Orally Available Approved Drugs against Ebola Virus In Vivo

**DOI:** 10.3390/microorganisms9030566

**Published:** 2021-03-10

**Authors:** Courtney L. Finch, Julie Dyall, Shuang Xu, Elizabeth A. Nelson, Elena Postnikova, Janie Y. Liang, Huanying Zhou, Lisa Evans DeWald, Craig J. Thomas, Amy Wang, Xin Xu, Emma Hughes, Patrick J. Morris, Jon C. Mirsalis, Linh H. Nguyen, Maria P. Arolfo, Bryan Koci, Michael R. Holbrook, Lisa E. Hensley, Peter B. Jahrling, Connie Schmaljohn, Lisa M. Johansen, Gene G. Olinger, Joshua T. Schiffer, Judith M. White

**Affiliations:** 1 Integrated Research Facility, Division of Clinical Research, National Institute of Allergy and Infectious Diseases, National Institutes of Health, Frederick, MD 21702, USA; courtney.finch@nih.gov (C.L.F.); dyallj@niaid.nih.gov (J.D.); elena.postnikova2@nih.gov (E.P.); janie.liang@nih.gov (J.Y.L.); huanying.zhou@nih.gov (H.Z.); dewaldl@ebsi.com (L.E.D.); michael.holbrook@nih.gov (M.R.H.); lisa.hensley@nih.gov (L.E.H.); jahrlingp@niaid.nih.gov (P.B.J.); connie.schmaljohn@nih.gov (C.S.); 2 Fred Hutchinson Cancer Research Center, Vaccine and Infectious Diseases Division, Seattle, WA 98109, USA; sxu3@fredhutch.org; 3 Department of Cell Biology, University of Virginia, Charlottesville, VA 22903, USA; en2b@virginia.edu; 4 National Center for Advancing Translational Sciences, Division of Preclinical Innovation, National Institutes of Health, Bethesda, MD 20892, USA; craigt@mail.nih.gov (C.J.T.); amy.wang@nih.gov (A.W.); xin.xu3@nih.gov (X.X.); Emma.Hughes@ucsf.edu (E.H.); patrick.morris@nih.gov (P.J.M.); 5 SRI International, Biosciences Division, 333 Ravenswood Avenue, Menlo Park, CA 94025, USA; jon.mirsalis@sri.com (J.C.M.); linh.nguyen@sri.com (L.H.N.); maria.arolfo@sri.com (M.P.A.); 6 Eurofins Panlabs, 6 Research Park Dr., St. Charles, MO 63304, USA; bryankoci@eurofins.com; 7 Emerging Viral Pathogens Section, National Institute of Allergy and Infectious Diseases, National Institutes of Health, Frederick, MD 21702, USA; 8 Zalicus Inc., Cambridge, MA 02142, USA; lmjohan@hotmail.com; 9 MRIGlobal, Gaithersburg, MD 20878, USA; golinger@mriglobal.org; 10 Department of Microbiology, University of Virginia, Charlottesville, VA 22903, USA

**Keywords:** filovirus, pandemic preparedness, synergy, viral pathogens, emerging viruses, mathematical modeling, projected benefit in humans, bepridil, sertraline, toremifene, apilimod

## Abstract

Outbreaks of Ebola ebolavirus (EBOV) have been associated with high morbidity and mortality. Milestones have been reached recently in the management of EBOV disease (EVD) with licensure of an EBOV vaccine and two monoclonal antibody therapies. However, neither vaccines nor therapies are available for other disease-causing filoviruses. In preparation for such outbreaks, and for more facile and cost-effective management of EVD, we seek a cocktail containing orally available and room temperature stable drugs with strong activity against multiple filoviruses. We previously showed that (bepridil + sertraline) and (sertraline + toremifene) synergistically suppress EBOV in cell cultures. Here, we describe steps towards testing these combinations in a mouse model of EVD. We identified a vehicle suitable for oral delivery of the component drugs and determined that, thus formulated the drugs are equally active against EBOV as preparations in DMSO, and they maintain activity upon storage in solution for up to seven days. Pharmacokinetic (PK) studies indicated that the drugs in the oral delivery vehicle are well tolerated in mice at the highest doses tested. Collectively the data support advancement of these combinations to tests for synergy in a mouse model of EVD. Moreover, mathematical modeling based on human oral PK projects that the combinations would be more active in humans than their component single drugs.

## 1. Introduction

The family *Filoviridae* contains human pathogens of high consequence including Ebola virus (EBOV), Sudan virus (SUDV) and Marburg virus (MARV) [1,2,3]. The largest outbreak of EBOV occurred in Western Africa between 2013 and 2016 leading to the deaths of over 11,000 individuals [2,4]. The second largest outbreak occurred in the northeastern region of the Democratic Republic of the Congo (DRC) between August 2018 and June 2020, and an eleventh outbreak recently occurred in the DRC (June to November 2020). There is now a licensed vaccine (Ervebo), a licensed therapeutic monoclonal antibody (mAb) (Ebanga), and a licensed therapeutic mAb cocktail (Inmazeb) against EBOV. These undoubtedly played significant roles in curbing the latest outbreaks. However, no vaccines or therapeutics are currently approved to prevent or treat human diseases caused by other members of the Ebolavirus genus (e.g., SUDV; Bundibugyo virus, Taï Forest Virus), by members of the Marburgvirus genus, nor for diseases that might be caused by emerging filoviruses (e.g., Reference [5]). We seek a pan-filovirus oral therapeutic that could be rapidly and economically deployed at the outset of new filovirus outbreaks. Such a therapeutic would mitigate disease during the time period (many months to years) when species- and/or isolate-specific therapeutics (e.g., therapeutic mAbs) and vaccines are being developed and deployed.

Towards the goal of developing a rapidly deployable low-cost oral pan-filovirus therapeutic, we and others have identified low molecular weight drugs approved for other indications that have activity against EBOV in cell cultures [6,7,8,9,10,11,12,13] (for recent reviews see [3,14,15,16,17,18]). A limitation of monotherapy drug repurposing as an anti-viral strategy is that often the maximum concentration of drug attainable (e.g., C_max_ in plasma) is below the effective dose range determined for the drug as an anti-viral agent in cell culture. Recognizing that, as well as the success of anti-viral drug cocktails against chronic human viral pathogens such as human immunodeficiency virus [19,20] and hepatitis C virus [21], we and other have identified pairs of approved drugs that block EBOV replication in cells synergistically [22,23,24,25,26]. Synergistic cocktails lower the doses of the component drugs needed and, reciprocally, increase anti-viral potency [27,28].

Our prior anti-EBOV drug synergy testing in cells [22] revealed several pairs of drugs that block EBOV synergistically including (bepridil + sertraline) and (sertraline + toremifene). Towards the goal of testing these combinations in a mouse model of EBOV disease (EVD), we identified a vehicle suitable for their administration by the oral route, demonstrated the efficacy of each drug formulated for oral delivery against EBOV in cells, determined the stability of the oral formulations over time, and conducted pharmacokinetic and tolerability studies that support the ability to conduct a test in mice to determine if these combinations act synergistically against EBOV in the mouse model. We also conducted pharmacokinetic/pharmacodynamics modeling studies, combined with a previously built EBOV viral dynamics model [29], which demonstrated that there could be potential benefits to administering these drug combinations to humans for treating EVD.

## 2. Materials and Methods

### 2.1. Ebolavirus (EBOV) Cell-Based Infection Assay

EBOV infection assays were performed essentially as described in References [22,30]. In brief, for in vitro single agent drug tests, Huh7 cells were plated at 30,000 cells/well in black clear bottom 96 well plates. 24 h later they were pretreated with drugs for 1 h and then infected with EBOV/Mak (Ebola virus/H.sapiens-tc/GIN/201 4/Makona-C05 (GenBank accession no. KX000398.1) (#IRF0165) at a moi of 0.21. The drugs were tested in 8-point dose response curves with 2-fold serial dilutions. Each dose was run in triplicate (*n* = 3), and each experiment was run on at least duplicate plates. After 48 h, the cells were fixed in formalin, and stained for the EBOV VP40 protein (mouse antibody #BMD04B007 A11) followed by peroxidase labeled goat α-mouse IgG (H+L), followed by a chemiluminescent substrate as described in Ref. [31]. Plates were then read using a Tecan plate reader (model M1000). For in vitro drug combination tests, cells, plated as above, were pretreated for 1 h prior to infection with a 6 × 6 matrix containing 2-fold serial dilutions of Drug 1 and Drug 2, and then assessed for effect on EBOV infectivity as described above for the single agent tests. For drug combincation experiments, cytotoxicity was determined (in parallel black opaque 96-well plates) using the Cell Titer-Glo Luminescent Cell Viability Assay (Promega, Durham, NC, USA), and each datapoint (infection and cell viability) was analyzed (in each experiment) with 3 replicates. All work with live EBOV was conducted in a BSL4 facility. Sources and catalogue numbers for the drugs tested are given in the Supplemental Document. Sources of cells and cell culture media components are given in Reference [32]. IC_50_ values were calculated as follows: background values were subtracted and inhibition was measured as percent relative to untreated infected cells. Non-linear regression analysis was then performed using GraphPad Software (La Jolla, CA, USA), and IC_50_ values were calculated from fitted curves (log [agonist] vs. response [variable slope] constrained to remain above 0. Error bars of dose–response curves represent the standard deviation (sd) of three replicates.

### 2.2. VSV-EBOV GP-Pseudovirus Infection Assay

VSV pseudoviruses encoding EBOV GP deleted for its mucin domain (GPΔ) were prepared as described in References [7,33,34], but using VSV helper virus deleted for its own G protein gene (VSVΔG) and encoding Renilla luciferase, VSV-ΔG-Luc (gift of Dr. Robert Doms, University of Pennsylvania). VSV-GPΔ-Luc infection assays were then performed essentially as described in References [7,33,34]. In brief, HEK293T/17 cells (ATCC CRL-11268) were plated in opaque white 96 well plates at a density of 30,000 cells/well. The cell culture medium employed was high glucose Dulbecco’s Modified Eagle Medium (DMEM) supplemented with 1% L-glutamine, 1% sodium pyruvate, and 1% antibiotic/antimycotic, all from Gibco Life Technologies (Carlsbad, CA, USA), and 10% supplemented calf serum (SCS; Hyclone, GE Healthcare Bio-Sciences, Pittsburgh, PA, USA). Eighteen hours later the cells were pretreated with the indicated concentrations of the indicated drug for 1 h and then infected with an amount of VSV-GPΔ-luc pretitered to yield ~100,000 relative light units in uninhibited samples. After 24 h, cells were lysed and analyzed for Renilla luciferase activity using the Renilla-Glo luciferase assay system from Promega (Durham, NC, USA) according to the manufacturer’s instructions. Each datapoint was analyzed (in each experiment) with 3 replicates. Sources and catalogue numbers for the drugs tested are given in the Supplemental Document.

### 2.3. Test of Apilimod against EBOV in the Mouse Model

The efficacy of Apilimod (Axon Medchem, Reston, VA, USA, cat. # 2500) was tested in the C57BL/6 mouse model of EVD. Apilimod was prepared fresh in saline (0.9% NaCl Injection, USP, Baxter Health Care Corporation, Charlotte, NC, USA) from powder each dosing day. Apilimod powder was weighed, aliquoted and stored at 4 °C one day prior to the study start.

Doses of 30 mg/kg or 44 mg/kg of apilimod mesylate (equivalent to 20.5 mg/kg or 30 mg/kg apilimod, respectively in 0.9% NaCl Injection, USP) were administered IP to female C57BL/6 mice (Charles River Laboratories, Frederick, MD, USA) once a day for 10 days, beginning ~4hr before exposure to ma-EBOV (mouse-adapted Ebola virus/Mayinga (GenBank accession no. KY425637.1). Compound efficacy was measured by assessing percent survival in the apilimod treated groups relative to the vehicle (saline) control group. Mice (10 per group) were challenged IP with 246 plaque forming units (PFU) of ma-EBOV on study day 0. Mice were observed for a total of 28 days, and mice that achieved a clinical score of 3 were euthanized.

Criteria for clinical scoring are outlined in the most current Integrated Research Facility (IRF) Animal-Care-and-Use-Committee-approved Animal Study Proposal. Mice were housed in an accredited BSL4 animal facility (accredited by the Association for Assessment and Accreditation of Laboratory Animal Care (AAALAC)). All animal procedures were approved (approval number IRF-032E, 22 January 2015) by the Animal Care and Use Committee of the National Institute of Allergy and Infectious Diseases, Division of Clinical Research, in compliance with the Animal Welfare Act regulations, Public Health Service policy, and the Guide for the Care and Use of Laboratory Animals recommendations.

### 2.4. Drug Formulation and Mouse Pharmacokinetic (PK) and Tolerability Tests

SRI Biosciences (Menlo Park, CA, USA) performed formulation and tolerability studies for bepridil, sertraline and toremifene. Details on the preparation of drugs in the 10 test vehicles analyzed are given in the Supplemental Document. The tolerability studies were performed with bepridil, sertraline and toremifene prepared in Vehicle 8 (V8, which is 80% PEG 400/20% of 0.1% Tween-20 in Water) on female C57BL/6 mice (Charles River Laboratories, Hollister, CA, USA) that were administered drugs orally (PO) for 11 consecutive says (once/day), followed by a 7-day recovery period.

Eurofins Scientific (St. Charles, MO, USA) performed the pharmacokinetic (PK) studies. In brief, bepridil-HCl, sertraline-HCl and toremifene citrate were prepared in V8 according to procedures outlined in the Supplementary Document. Single oral doses of bepridil (150 and 500 mg/kg), sertraline (30 and 60 mg/kg) and toremifene (100 and 200 mg/kg) were administered to female C57BL/6 mice (provided by BioLasco Taiwan, a Charles River Laboratories Licensee) and analyzed for the indicated parameters according to standard Eurofins procedures. All aspects of the work, including housing, experimentation, and disposal of animals were performed in general accordance with the Guide for the Care and Use of Laboratory Animals: Eighth Edition (National Academy Press, Washington, DC, USA, 2011) in an AAALAC-accredited laboratory animal facility. The animal care and use protocols were reviewed and approved by the respective IACUCs at Pharmacology Discovery Services Taiwan, Ltd. (approval number PK001-09212018, 21 September 2018) and SRI International (approval number 02006, 3 November 2016).

### 2.5. Mathematical Modeling Studies

#### 2.5.1. Human Pharmacokinetic Data

Mean bepridil PK data were extracted from Reference [35] using WebPlotDigitizer (WebPlotDigitizer—Extract data from plots, images, and maps, Pacifica, CA, USA), where 5 healthy male volunteers (20~49 years old) were administered 400 mg bepridil-HCl orally. Mean toremifene PK data were extracted from reference [36] using the same extraction method, where volunteers were orally administered 50 mg (*n* = 3) and 100 mg (*n* = 3) toremifene. Sertraline PK data were gathered from reference [37], containing raw data for 14 healthy male volunteers (28~40 years old) given a single oral dose of 100 mg sertraline (Teva-Sertraline 100 mg, Sertraline HCl).

#### 2.5.2. Pharmacokinetic Modeling of Bepridil, Sertraline and Toremifene

**Figure 1:**
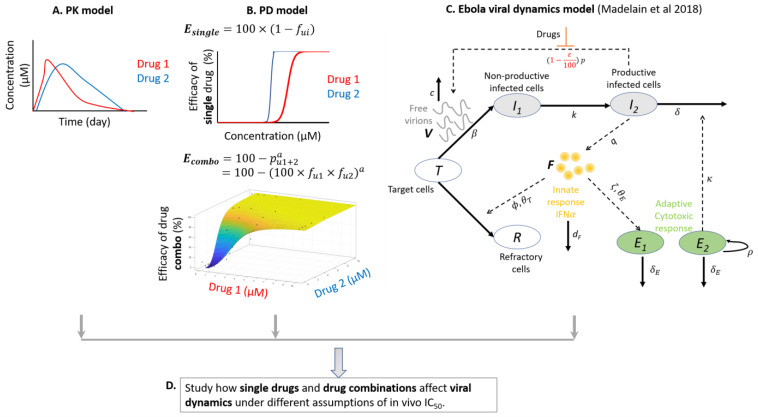
Frameworks for studying the advantage of drug combinations against EBOV infection. (**A**) PK modeling (drug concentration in human plasma or serum over time). (**B**) PD modeling (drug concentration dependent inhibition of EBOV replication) of single drugs (top) and drug combinations (bottom). (**C**) EBOV dynamics model (plasma viral load over time in cynomolgus macaques, schematic styled after Figure 3 in [29]). (**D**) The PK, PD (single drugs and drug combinations), and EBOV viral load dynamics model, are combined under different assumptions of in vivo IC_50_s (the plasma concentration of drug required to inhibit EBOV replication by 50%, the true in vivo drug potency against EBOV, in humans) to predict EBOV viral load trajectories in humans in the presence of single drugs or drug combinations. Panel C was adapted from Reference [29]. See text for sources of input data.

To model the PK of bepridil, sertraline and toremifene (Figure 1A), we fitted PK data (as mentioned above) to available PK models in Monolix [38]. We selected the best PK model for each drug that has the lowest Aikaike Information Criteria (AIC) score (Supplemental Figures S1–S3 and Supplemental Tables S1–S3). AIC rewards for close fit to data and penalizes for unnecessary complexity.

#### 2.5.3. Pharmacodynamic (PD) Modeling of Single Drugs and Drug Combinations

PD data of single drugs, bepridil, sertraline, and toremifene, as well as drug combinations of (sertraline + bepridil) and (sertraline + toremifene), were gathered from [22]. The in vitro IC_50_s and hill coefficients of bepridil, sertraline, and toremifene applied alone are from Reference [22] and are reported in Supplemental Table S4. The efficacy of a single drug (%) equals [20]:

(1) $$E_{single}\;=\;100*\left(1-f_{ui}\right)\;$$

where *f_ui_* is the fraction of infection events unaffected by drug *i*, that:

$$f_{ui}\;=\;\frac{1}{1+{\left(\frac{D_{i}}{IC_{50,\;i}}\right)}^{m_{i}}}$$

$D_{i}$ is the concentration of drug *i* (μM). $IC_{50,\;i}$ and $m_{i}$ are the concentration of drug *i* (μM) required for 50% inhibition and the hill coefficient of drug *i* when drugs were used alone.

To model the efficacy of drug combinations, we modified a pre-existing Bliss independence model [20] by adding a power factor, *a*, to the percentage of infection events unaffected by both drugs (*p*_*u*1+2_), in order to capture the possible synergistic effects of drug combinations (Figure 1B):

(2) $$E_{combo}\;=\;100-p_{u1+2}^{a}\;=\;100-{\left(100\times f_{u1}\times f_{u2}\right)}^{a}\;$$

Parameters of $IC_{50,\;i}$ and $m_{i}$ in the expression of *f_ui_* were re-estimated in drug combination assays.

We previously defined in vivo IC_50_ as the plasma drug concentration at which viral replication is inhibited by 50% in vivo [39,40]. Since in vivo IC_50_s of bepridil, sertraline and toremifene in humans in single drug treatments and combined drug treatments are unknown, we made various assumptions based on in vitro IC_50_s measured in the dose response assays for the single drugs and drug combinations. Specifically, we assumed that in vivo IC_50_s are 0.1×, 1×, 5× and 10× of empirically observed in vitro IC_50_s, and then projected pharmacodynamics of single drugs (Supplemental Figure S4) and drug combinations (see Section 3.5.1). PK and PD models were then linked to estimate percent of viral replication events inhibited as a function of time.

#### 2.5.4. Mathematical Modeling of EBOV Viral Dynamics

To study the possible advantage of drug combinations against EBOV infection, we used a previously built EBOV viral dynamics model [29], which is composed of viral production-inhibition, a cell defense mechanism mediated by IFNα, innate immune response and adaptive immune response (Figure 1C).

We assume that drugs impact viral replication in the model according to drug plasma/serum concentration and PD curve according to the equation:

(3) $$\frac{dV}{dt}\;=\;\underset{\begin{array}{c} Inhibition\\ of\;viral\;replication\\ by\;drug \end{array}}{\underbrace{\left(1-\frac{\varepsilon }{100}\right)}}\times \underset{\begin{array}{c} Replication\\ of\;virions \end{array}}{\underbrace{pI_{2}}}-\underset{\begin{array}{c} clearance\\ of\;virions \end{array}}{\underbrace{cV}}\;$$

where ε is the efficacy of a single drug (*E_single_*, %) or the efficacy of a drug combination (*E_combo_*, %), which is a function of the plasma/serum concentration of a drug (Equation (1)) or drugs (Equation (2)). Then, (1 − ε/100) describes the fraction of inhibition on viral replication (*pI_2_*) from reproductively infected *I_2_* cells, where *p* stands for production rate of virions (*V*). Virions have a clearance rate *c*. Thus, Equation (3) is the mathematical expression of the rate change of viruses (*V*).

Therefore, the EBOV viral dynamics model (Figure 1C) consists of a system of ordinary differential equations [29]:

$$\begin{array}{c} \frac{dT}{dt}\;=\;-\beta TV-\frac{\phi TF}{F+\theta_{T}}\\ \frac{dI_{1}}{dt}\;=\;\beta TV-kI_{1}\\ \frac{dI_{2}}{dt}\;=\;kI_{1}-\delta I_{2}-\kappa I_{2}E_{2}\\ \frac{dR}{dt}\;=\;\frac{\phi TF}{F+\theta_{T}}\\ \frac{dV}{dt}\;=\;\left(1-\frac{\varepsilon }{100}\right)I_{2}p-cV\\ \frac{dF}{dt}\;=\;qI_{2}-d_{F}F\\ \frac{dL}{dt}\;=\;q_{L}I_{2}-d_{L}F\\ \frac{dN}{dt}\;=\;q_{N}I_{2}-d_{N}F\\ \frac{dE_{1}}{dt}\;=\;\sigma -\frac{\zeta FE_{1}}{F+\theta_{E}}-\delta_{E}E_{1}\\ \frac{dE_{2}}{dt}\;=\;\rho E_{2}\left(1-\frac{E_{2}}{E_{0}}\right)-\delta_{E}E_{2} \end{array}$$

## 3. Results

### 3.1. Selection of Drugs for Combination Tests in Mice

We previously reported on 78 pairwise combinations of approved drugs against EBOV in Huh7 cells [22]. Seven highly synergistic combinations are listed in Table 1. We chose to pursue the second (bepridil + sertraline) and fourth (sertraline + toremifene) pairs for the following reasons. We excluded the top pair (aripiprazole + piperacetazine) because piperacetazine is now only licensed for veterinary use in the United States of America (USA) and because in a first test (aripiprazole + piperacetazine) caused hypersomnolence in mice infected with EBOV, and thus malnutrition and dehydration likely contributed to the low (10%) survival rate [7]. We excluded the third pair (sertraline + clomiphene) because of poor human plasma exposure following oral administration of clomiphene (Supplemental Table S5) and variable survival rates in mice treated with clomiphene as a single agent (Supplemental Table S6). We also chose to exclude combinations ranked as numbers 5–7 (apilimod paired with clomiphene, azithromycin, or toremifene). Although apilimod has potent activity against EBOV in cell cultures [32,41,42] and has a human C_max_/IC_50_ >1 (Supplemental Table S5) suggesting potential activity in vivo, in a first test as a single agent in female C57BL/6 mice challenged with mouse-adapted EBOV (ma-EBOV), apilimod provided no survival advantage (Supplemental Figure S5). This was likely because apilimod is known to inhibit IL12/23 production [43,44], thereby interfering with IL12-mediated inhibition of EBOV infection (via interferon γ production) in macrophages [45], which are key targets of EBOV infection [2]. Hence, the components chosen for pairwise tests in mice were bepridil, sertraline and toremifene in the combinations (bepridil + sertraline) and (sertraline + toremifene).

Bepridil, sertraline and toremifene are orally available drugs (Supplemental Table S5) that block infections by EBOV and MARV in vitro [6,7,9]. Sertraline (Zoloft) and toremifene (Fareston) are prescribed in the USA and abroad for depression and metastatic breast cancer, respectively. While in use in several countries, Bepridil (Vascor) has been withdrawn as an antihypertensive in the USA. All three drugs showed efficacy via the intraperitoneal (IP) route in a mouse model of EBOV infection [6,7] (also see Supplemental Table S6), and bepridil, which showed 100% protection in the mouse model [7], also strongly protected female Balb/c mice challenged with 195 PFU of ma-MARV Angola [47]. Sertraline was not effective at the dose employed in a test in rhesus macaques challenged with 1090 PFU of EBOV/Mak-C05 [48] (see Discussion). Bepridil, sertraline and toremifene block EBOV entry into cells through late endosomes (LE) that bear the EBOV receptor, Niemann-Pick C1 (NPC1) [49,50,51,52,53]; they do not block EBOV binding to host cells, internalization from the cell surface or trafficking to NPC1^+^ LE [6,7,54]. Two co-functioning mechanisms likely account for their anti-EBOV action: (i) binding to a pocket in EBOV GP [55,56], with consequent effects on GP interaction with NPC1 [54] and conformational changes needed to elicit fusion [55,56,57], as well as (ii) disruptive effects on the composition and function of LEs [34,54]. Bepridil has also been reported to bind to two-pore calcium channel 2 (TPC2) [58], a host LE protein required for EBOV entry [59].

### 3.2. Selection of Drug Doses for Combination Tests in Mice

For combination tests we aim to achieve doses resulting in 20–25% survival as single agents when given orally to mice enabling assessment of whether administration in pairs results in greater than 50% survival (i.e., more than an additive effect). We therefore chose top doses of each drug (Table 2) that should be safe in mice (based on LD_50_) and achieve ≥50% survival, contemplating down-dosing (following a dose-down study) to achieve 20–25% survival as single drugs. The reasonings for the top dose of each drug are as follows (see Supplemental Tables S5 and S6 for details). Bepridil: The aim is to achieve a C_max_ of ~4 μM (average IC_50_, Supplemental Table S5) to yield greater than or approximately equal to 50% survival. An oral dose (PO) of 150 mg/kg yielded a C_max_ of 1.5 μM [35]. Hence, 2.67-fold (4/1.5) more bepridil would be needed, suggesting a top oral dose of 400 mg/kg. As the oral LD_50_ is 2069 mg/kg, we opted for a top dose of 500 mg/kg. We note that the C_max_ for the IP dose (12 mg/kg) of bepridil that yielded 100% protection was 3.3 μM (P. Glass, personal communication). Sertraline: 10 mg/kg sertraline IP on a BID schedule (twice daily dosing) yielded 70% survival (Supplemental Table S6). We approximated that daily dosing with 30 mg/kg would yield a similar level of protection. We found that the oral C_max_ of sertraline (prepared in 0.9% saline) was ~½ that obtained via the IP route (Table 2). Hence, we predicted that 30 mg/kg PO should yield ~35% survival. Given an oral LD_50_ of 336 mg/kg we chose 60 mg/kg as the top dose. Toremifene: 60 mg/kg toremifene (IP) on a QOD schedule (d0, d1 and then every other day for a total of six doses) yielded 50% survival. We approximated that 30 mg/kg SID (once daily) would also protect ~50% of infected mice. A comparison of PO (in 0.9% saline) to IP (in DMSO) administration indicated that the C_max_ for oral sertraline is 1/7 that seen via the IP route (Table 2). We therefore reasoned that 30 mg/kg PO SID would yield ~7% survival indicating a need for ~7-fold higher dosing. Based on the oral LD_50_ of 3000 mg/kg, we opted for 200 mg/kg as the top toremifene dose (estimated to yield ~50% survival).

### 3.3. Oral Formulation, Stability and Activity Tests of Bepridil, Sertraline and Toremifene

The next step was to identify a vehicle compatible for PO delivery of all three drugs. For this purpose, we tested the solubility of bepridil, sertraline and toremifene in 10 vehicles. As seen in Supplemental Table S7, none of the drugs were soluble in aqueous solution, but all three were soluble in Vehicles (V) 7–9. For further studies we adopted V8: 80% PEG 400/20% of 0.1% Tween-20 in water. We chose V8 because it reproducibly formed a clear solution with all of the three test drugs and because it is comprised of more commonly available reagents (as compared with V7 and V9). Details for the preparation of V1-10 and stock solutions of bepridil, sertraline and toremifene are given in the Supplemental Document. As noted there, Bepridil requires resuspension following storage in V8 at 4 °C or RT.

**Figure 2:**
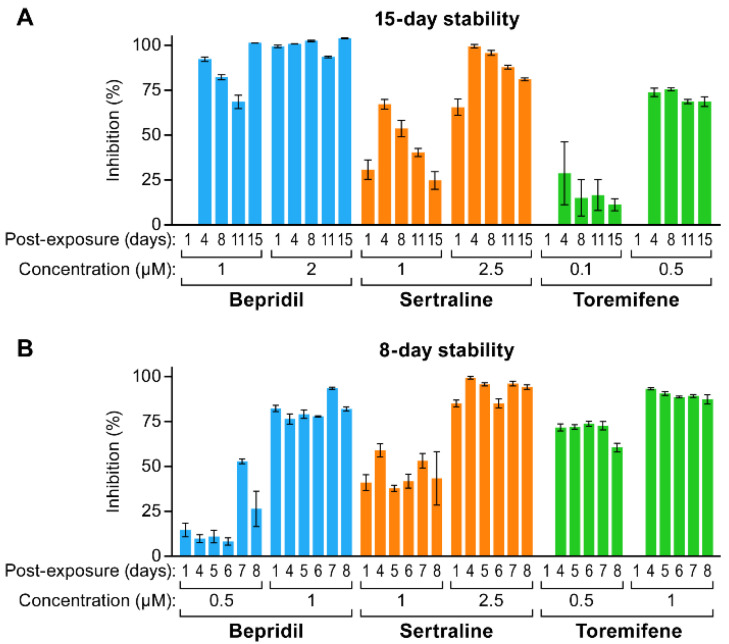
Activity of bepridil, sertraline and toremifene in V8 against VSV-EBOV-GPΔ-Luc over time. Bepridil-HCl (50 mg/mL), sertraline-HCl (6 mg/mL), and toremifene citrate (8.5 mg/mL) were prepared in V8 on day 1 (d1) as described in the Supplementary Document, and stored at 4 °C for (**A**) 15 or (**B**) 8 days. On the indicated day the stock was brought to RT. HEK293T/17 cells were pretreated with drugs as indicated, infected with VSV-EBOV-GPΔ-Luc, and scored for infection as described in the Methods section. All samples were analyzed in triplicate, and the data are averages +/− sd.

**Figure 3:**
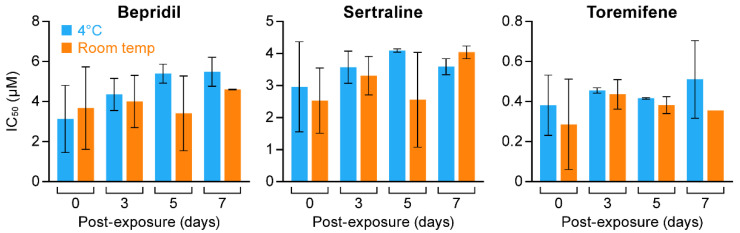
Activity of bepridil, sertraline and toremifene in V8 against EBOV/Mak over seven days. Drug stocks were prepared on d0 in V8 as for Figure 2, stored at 4 °C (blue bars) or RT (orange bars), and then tested in 8 point dose reScheme 0. d5, and d7 using 2-fold serial dilutions in V8. Huh7 cells were pretreated with the indicated drugs and infected with EBOV/Mak (moi 0.21). Two experiments were conducted; for each, dose response curves were run on duplicate plates with 3 replicates of each dose on each plate, and analyzed to obtain IC_50_s as described in the Methods section. Data from the triplicate technical replicates on each plate were averaged. The data shown are the averages of the average IC_50_s +/− sd from two to four plates. Data from plates with Z’ factor <0.2 were not included.

We next tested the stability of bepridil, sertraline and toremifene in V8. We first did this using VSV pseudovirions bearing EBOV GPΔ (GP deleted for its mucin domain) and encoding Renilla luciferase (Luc), a system that can be used under BSL2 conditions to monitor EBOV entry [33,60,61]. In a first test, stock solutions in V8 were prepared and stored at 4 °C for 1–15 days. HEK293/T17 cells were pre-treated each day for 1 h with the indicated concentration of the indicated drug. Cells were then infected with VSV-EBOV-GPΔ-Luc and infection scored 24 h later based on the Luc reporter. As seen in Figure 2A, whereas bepridil maintained inhibitory activity over 15 days, sertraline and toremifene began to lose activity after ~1 week. In a second experiment, all three drugs appeared stable in V8 over an 8-day period with possible small losses of activity between days 7 and 8 (Figure 2B). We extended these studies to authentic EBOV (Makona isolate; EBOV/Mak), testing drugs in V8 stored at either 4 °C or RT for seven days. As seen in Figure 3, all of the drugs were stable for 7 days, with possible small loss of potency (<2-fold) for Bepridil stored at 4 °C.

**Figure 4:**
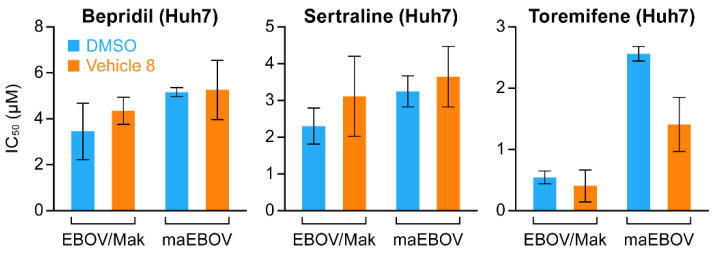
Activity of bepridil, sertraline and toremifene in DMSO and V8 against EBOV/Mak and ma-EBOV. Drug treatments and infections were conducted as described in the Methods section and legend to Figure 3. The data shown are the average IC_50_s +/− sd from four plates excepting one plate with Z’ factor <0.2.

We then assessed the potency of drugs stored for seven days at RT against EBOV/Mak and ma-EBOV, as the latter would be used for efficacy tests in mice. Similar potencies were seen for all three drugs whether prepared in DMSO or V8. Lower activity was noted for toremifene against ma-EBOV compared to EBOV/Mak, but this was seen for toremifene prepared in both DMSO and V8 (Figure 4).

### 3.4. Pharmacokinetic and Tolerability Tests of Reformulated Bepridil, Sertraline and Toremifene

Having demonstrated solubility and defined stability of bepridil, sertraline and toremifene in V8, we conducted pharmacokinetic (PK) analyses in female C57BL/6 mice administered the drugs (in V8) by oral gavage. For each drug, the planned top dose and one lower dose were tested. The C_max_, t_1/2_ and mean residence time (MRT) are given in Table 3, along with average IC_50_s and consequent C_max_/IC_50_ values. The pre-PK predictions for C_max_ at the top doses (based on considerations in Section 3.2) were: bepridil ~3 μM, sertraline ~1.2 μM, and toremifene ~4.4 μM. The C_max_ values obtained (at the top doses) were near the predictions: ~1.8- and 1.2-fold higher for bepridil and sertraline, respectively, and ~1.4 fold lower for toremifene.

**Figure 5:**
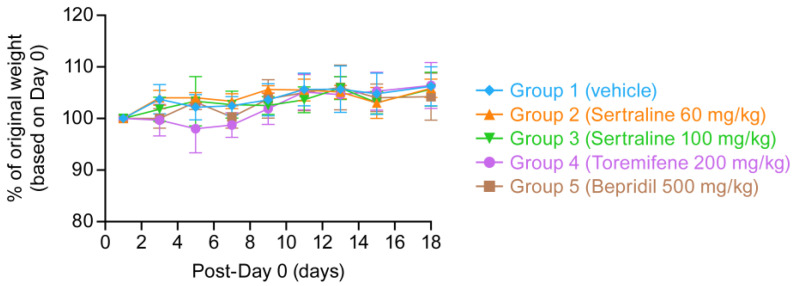
Bepridil, sertraline and toremifene (in V8) are well-tolerated by mice. Female C57BL/6 mice (5/group) were administered the indicated doses of bepridil, sertraline and toremifene in V8 by oral gavage for 11 consecutive days. Daily average weights for 18 days for each group are shown +/− sd.

We, therefore, tested the tolerability of the three drugs in V8, given once daily PO, for eleven consecutive days (drugs were freshly prepared in V8 every 5 or 7 days and stored at RT) in female mice that were weighed and observed for a total of 18 days. Bepridil and toremifene were administered at the top planned doses (500 and 200 mg/kg, respectively); sertraline was administered at the top planned (60 mg/kg) and one higher (100 mg/kg). As seen in Figure 5, no aberrations in weight gain were seen. All mice survived, and no toxicologically significant changes were seen. Hence, bepridil, sertraline and toremifene are well tolerated in mice at the top target doses.

### 3.5. Mathematical Modeling Studies to Predict In Vivo Efficacy of Drug Combinations in Humans

#### 3.5.1. Pharmacodynamic (PD) Modeling of Drug Combinations

**Figure 6:**
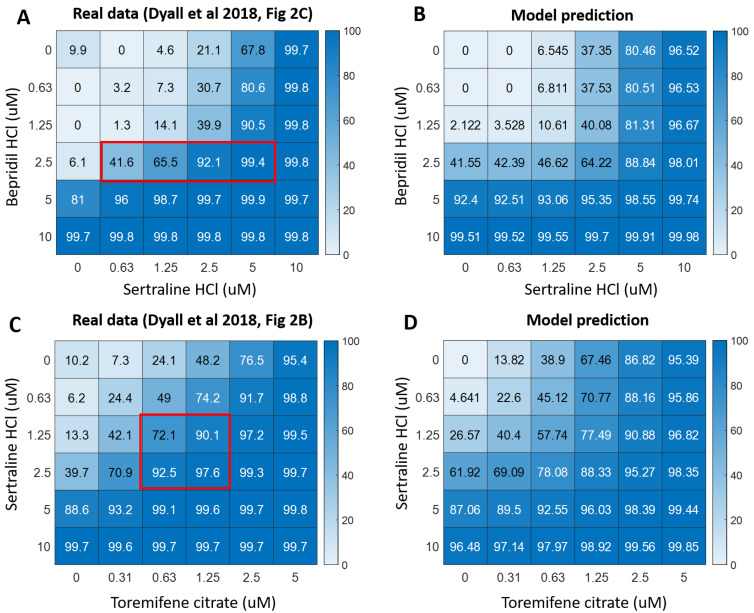
Empirical and projected efficacies of drug combinations. (**A**,**C**) Empirically quantified efficacies of (bepridil HCl + sertraline HCl) and (sertraline HCl + toremifene citrate) in Huh7 cells [22]. Highly synergistic regions are highlighted in red boxes, which have ∆Bliss values <−0.3. (**B**,**D**) Model-projected efficacies of (bepridil HCl + sertraline HCl) and (sertraline HCl + toremifene citrate) using the Bliss model. Colors in each heatmap represent the percentage of inhibition, with 0 for no inhibition and 100 for complete inhibition of EBOV replication.

We developed a pharmacodynamic (PD) model to project the in vivo efficacy of drug combinations in humans. For combinations of (sertraline + bepridil) and (sertraline + toremifene), the in vitro IC_50_s and Hill coefficients for each drug, as well as the power factors which assess synergy (a), are provided in Table 4. The high R^2^ (>0.93), indicates good model fit to the data. Hill coefficients exceeding 1 denote a steep dose response relationship and a~1 indicates limited synergy on average across all combinations of drug levels. The good fit provided by the Bliss model implies potent multiplicative drug effects. As the model does not capture the regions of high synergy noted in reference [22] (also see Figure 6A,C), its projection of in vivo potency at certain drug level combinations represents a minimum projection of potency (see Section 2.5 for Definitions of Terms and Details).

We visually compared the empirical in vitro data from [22], displayed in (Figure 6A,C), and our model predictions for human in vivo efficacy under the condition where in vivo efficacy equals in vitro efficacy (Figure 6B,D). For both combinations, our model provides good predictions of efficacies when both drugs are present, but somewhat poorer predictions when only one drug is present (compare heat maps for top row and left-most column in Figure 6B,D vs. Figure 6A,C). This does not affect our results when investigating the effects of single drugs and drug combinations on EBOV viral load dynamics, because when only one drug is present, we simulated the efficacy of the single drug using the PD model for the single drug (Equation (1)) and parameters provided in Supplemental Table S4. Further, our model underestimated synergy in highly synergistic regions (indicated with red boxes in Figure 6A,C), and hence our projected efficacies are lower than empirical efficacies.

#### 3.5.2. Projections of PK, PD, and EBOV Viral Dynamics in Humans Treated with Sertraline, Bepridil or Their Combination

**Figure 7:**
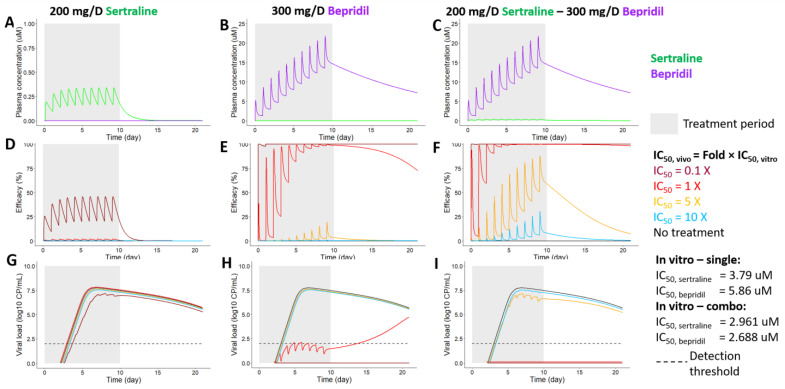
Projections of pharmacokinetics (PK), pharmacodynamics (PD), and EBOV viral load dynamics when sertraline and bepridil are used alone and together. Treatment periods start on day 0 and end on day 10, as indicated with gray backgrounds. Doses are 200 mg/day for the single sertraline treatment (**A**,**D**,**G**), 300 mg/day for the single bepridil treatment (**B**,**E**,**H**), and 200 mg/day sertraline—300 mg/day bepridil for the treatment of the drug combination (**C**,**F**,**I**). We assumed various in vivo IC_50_s in each treatment, which are shown in colors of sky blue for 10×, orange for 5×, red for 1× and dark red for 0.1× of in vitro IC_50_s (panels **D**–**I**). *(***A**–**C**) PK projections of sertraline (in green) and bepridil (in purple) and their combination. Note: The concentration of sertraline equals 0 µM over time in the single bepridil therapy (**B**), while it is greater than 0 µM (~0.25 µM) during the treatment period (gray shaded region) in the drug combination therapy (**C**). *(***D**–**F**) Projected efficacies of single sertraline, single bepridil, and their combination under assumptions of various in vivo IC_50_s. (**G**–**I**) Model-simulated EBOV viral load dynamics (log_10_(Cp/mL), log_10_(copy number/mL)) under different treatment regimens: single sertraline, single bepridil and their combination, when in vivo IC_50_s were varied. In graphs **G**–**I**, in cases where curves overlap (e.g., for the 5× and 10× simulations of in vivo IC_50_s), the lines were shifted just enough to better visualize the predicted viral loads. Note: a straight line at zero (e.g., in panels **H**,**I**) indicates a lack of detectable remaining virus.

We combined PK, PD and EBOV viral dynamic models (Figure 1) to sertraline and bepridil alone and together, to study if the drug combination provides more protection against EBOV infection than the components as single drug treatments. Treatments started on day 0, representing a post exposure prophylaxis scenario where people have had close contact with an infected patient, but do not yet have symptoms. With 10 days of 200 mg/day sertraline treatment, 10 days of 300 mg/day bepridil treatment, and 10 days of combined treatment (200 mg/day sertraline and 300 mg/day bepridil), we projected the plasma concentration of sertraline and bepridil over time (Figure 7A–C).

We further simulated the percentage of viral replication inhibited over time under each treatment assuming different in vivo IC_50_s (Figure 7D–F), which were set at 0.1, 1, 5, and 10 times the in vitro IC_50_s. This is because true in vivo IC_50s_ (the plasma concentration of drug required to inhibit replication by 50% in vivo) of a drug used alone or in combinations are unknown. Our past work on antiviral therapies demonstrated that the in vivo IC_50_ may be 5–10 fold higher than in vitro estimates thereby necessitating higher drug peaks and troughs [39,40].

Sertraline alone limited only ~30% of EBOV viral replication even when the in vivo IC_50_ was assumed to be much lower (0.1×) than the in vitro IC_50_ estimated from the dose response (D) of the single drug, with a ~0.5–1.0 log consistent reduction in viral load (Figure 7G). Bepridil completely prevented viral replication and infection when the in vivo IC_50_ was modeled to be 0.1X the in vitro IC50 (Figure 7E,H). Moreover, when the in vivo IC_50_ of bepridil was assumed to equal its in vitro estimate, it also suppressed viral load through day 14. With the drug combination (sertraline + bepridil), the efficacy is boosted such that anti-viral efficacy is seen even when the in vivo IC_50_ was assumed to be 1X and 5X the in vitro IC_50_ estimate from drug combination assays (Figure 7F,I). Thus, the combination of sertraline and bepridil may provide better protection against EBOV infection compared with treatments with either sertraline alone or bepridil alone, when the human in vivo IC_50_ is 1X or even 5X of the in vitro IC_50_.

#### 3.5.3. Projections of PK, PD, and Ebola Viral Dynamics in Humans Treated with Sertraline, Toremifene or Their Combination

**Figure 8:**
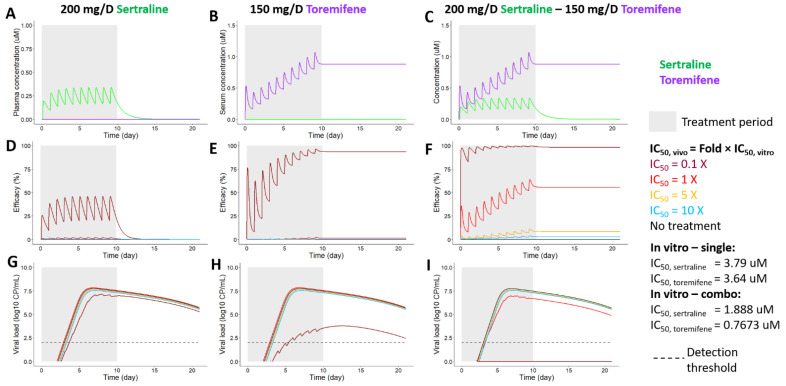
Projections of pharmacokinetics (PK), pharmacodynamics (PD), and EBOV viral dynamics when sertraline and toremifene are used alone and together. Treatment periods start on day 0 and end on day 10, as indicated with gray backgrounds. Doses are 200 mg/day for the single sertraline treatment (**A**,**D**,**G**), 150 mg/day with a loading dose of 300 mg/day (on day 0) for the single toremifene treatment (**B**,**E**,**H**), and 200 mg/day sertraline—150 mg/day toremifene with a loading dose of 300 mg/day (on day 0) for the treatment of the drug combination (**C**,**F**,**I**). We assumed various in vivo IC_50_s in each treatment, which are shown in colors of sky blue for 10×, orange for 5×, red for 1× and dark red for 0.1× of in vitro IC_50_s (**D**–**I**). (**A**–**C**) PK projections of sertraline (in green) and toremifene (in purple). (**D**–**F**) Projected efficacies of single sertraline, single toremifene, and their combination under assumptions of various in vivo IC_50_s. (**G**–**I**) Model-simulated EBOV viral load dynamics (log_10_(Cp/mL), log_10_(copy number/mL)) in different treatments: single sertraline, single toremifene and their combination, when in vivo IC_50_s were varied. In graphs **G**–**I**, in cases where curves overlap (e.g., for the 5× and 10× simulations of in vivo IC_50_s), the lines were shifted just enough to better visualize the predicted viral loads. Note: a straight line at zero (e.g., in panel **I**) indicates a lack of detectable remaining virus.

Similarly, we investigated whether the combination of (sertraline + toremifene) may be more efficient in limiting EBOV viral replication than single drug therapy, by combining PK, PD and Ebola viral dynamic models (Figure 8). Treatments started on day 0 and continued for 10 days. We projected the concentration of sertraline and toremifene over time in plasma or serum, respectively, with doses of 200 mg/day for sertraline (Figure 8A), 150 mg/day of toremifene with a 300 mg/day loading dose on day 0 (Figure 8B), and their combination (Figure 8C). We further simulated the percentage of viral replication eliminated by each treatment assuming different values for in vivo IC_50_s (Figure 8D–F) as described above for the combination (bepridil + sertraline). Sertraline as a single agent is discussed in the previous section with data from Figure 7A,D,G replotted in Figure 8A,D,G.

Toremifene alone was more efficient in limiting viral replication compared with sertraline alone when the in vivo IC_50_ was assumed to be much lower (0.1×) than the in vitro IC_50_ estimated in the dose response curve for the single drugs (Figure 8E,H vs. Figure 8D,G). However, neither sertraline alone nor toremifene alone protected against viral replication when the in vivo IC_50_ was assumed to equal the in vitro values (i.e., for 1× in Figure 8D,E,G,H, red curves). In contrast, the combination of (sertraline + toremifene) effectively increased the efficacy of the combination and eliminated viral replication with high potency when the in vivo IC_50_ was assumed to be both 0.1× and 1× the in vitro IC_50_ (Figure 8F,I; dark red and red curves). These findings suggest that (sertraline + toremifene) may provide better inhibition of viral replication than corresponding single drug therapies.

## 4. Discussion

Filoviruses from the Ebolavirus and Marburgvirus genera cause life-threatening diseases of global concern. While an effective vaccine and two monoclonal antibody therapies have recently been approved for the management of disease caused by Ebola ebolavirus (EBOV), neither vaccines nor therapies are available for other consequential filoviruses including Sudan, Bundibugyo, and Taï Forest viruses, Marburgvirus, nor for potential emergent filoviruses [2,3,4,5]. We contend that there remains a pressing need for shelf-ready low-cost oral therapeutics that could be easily distributed and administered to mitigate disease at the earliest identification of a disease-causing filovirus, especially in resource-challenged regions around the world. We posit that a cost-effective treatment will include a synergistic combination of orally available approved drugs. Synergistic combinations are favored over monotherapies due to their dose-lowering abilities and reduced chances of generating resistant viral strains [19,21,27,28]. Moreover, while two low molecular weight drugs appeared promising as intravenous monotherapies in non-human primate (NHP) models [62,63,64], two others provided by the oral route did not [48,65], and no small molecule monotherapies have yet proven highly effective against EBOV disease (EVD) in humans [66].

Towards the goal of developing a cocktail of orally available approved drugs with which to treat EVD patients, several laboratories have identified combinations of approved drugs that synergistically inhibit EBOV in cell cultures [22,23,24,25,26]. However, to date there has been no clear report of drug synergy against EBOV in an animal model. Based on our prior work [22] and addition tests (Table 1 and Supplemental Table S8), we advocate tests of (bepridil + sertraline) and (sertraline + toremifene) in the mouse model of EVD. Our rationale for these drug pairs is given in Section 3.1. Pursuing those choices, we identified a common solubilizing vehicle for oral delivery and demonstrated drug stability and efficacy upon storage. Based on pharmacokinetic (PK) and tolerability tests, we provide evidence that it should be possible to assess if (bepridil + sertraline) and/or (sertraline + toremifene), given by oral gavage, synergistically increase survival in mice challenged with EBOV. We also provide a modeling study demonstrating that both drug pairs are projected to function synergistically in humans.

Bepridil, sertraline and toremifene have all shown survival benefits in the mouse model of EVD as single agents (Table 2 and Supplemental Table S6) (also see [6,7]), and the combinations of (bepridil + sertraline) and (sertraline + toremifene) were found to be synergistic against EBOV in Huh7 cells (Table 1) (also see [22]). From data in Dyall 2018 [22], efficacies for the three drugs were enhanced ~2 to 4-fold. In mice, the oral C_max_/IC_50_ for the drugs at the top doses proposed for initial testing, which are well-tolerated (Figure 5), were calculated to be 1.18, 0.56, and 1.74, respectively, for bepridil, sertraline and toremifene (Table 3). Assuming ~3-fold dose reductions when used in combinations all of the drugs should yield mouse plasma exposures greater than their respective IC_50_ values (well above for bepridil and toremifene). Hence, we posit that the proposed top oral doses should yield significant protection and that, perhaps requiring lower doses following a dose-down study, it will be possible to attain survival levels of 20–25%, thus enabling tests of whether either of these drug combinations functions synergistically in mice. All three drugs protect against multiple species of Ebolavirus and two species of Marburgvirus in cell cultures [6,7] and, in addition to strongly protecting mice against EBOV (Table 2 and Supplemental Table S6) [6,7], bepridil has provided strong protection in a mouse model of marbugvirus disease [47]. Hence, there is potential that the proposed combinations would be pan-filoviral.

Regarding sertraline, we are aware that it did not protect macaques when given orally as a single agent [48]. In the cited study the dose was 200 mg/day (in treat tablets), which represented a mean daily dose of 55 mg/kg. In the reported retrospective PK test of a single dose (50 mg/kg) of sertraline in uninfected macaques, the C_max_ was 179 ng/mL (0.58 μM) [48], which is ~4.5-fold below the average IC_50_ for sertraline against EBOV in liver cells in vitro (Supplemental Table S5). The in vivo IC_50_ of sertraline (in infected macaques) may be even higher. Our simulations suggest the possibility of a potent antiviral effect of the combination (bepridil + sertraline) even if the in vivo IC_50_ in humans is 1× or 5× of the in vitro IC_50_, with anti-viral activity seen even if the in vivo IC_50_ is 10× the in vitro IC_50_ (Figure 7F). Collectively these findings suggest a need for a higher dose of sertraline to curb EBOV infections, and we propose that the effectiveness of sertraline would be boosted in a combination, for example with bepridil. Similar reasoning supports a focus on drug combinations in drug repurposing efforts for diseases caused by other high consequence viral pathogens.

Our modeling studies (Figure 7 and Figure 8) indicate that the combinations of (bepridil + sertraline) and (sertraline + toremifene) could deliver the additional potency required for a clinically meaningful decrease in EBOV viral load, depending on the actual in vivo IC_50_. Indeed, in a retrospective analysis, viral loads in EVD survivors were found to be ~1 log lower than in non-survivors [67,68], which is comparable to what our model projects for certain scenarios.

The next steps are to do a dose down study of bepridil, sertraline and toremifene in V8 in mice challenged with EBOV, starting with the top doses proposed in Table 3. Once the ~20–25% survival doses are determined the stage would be set to perform combined oral PK studies and to test if the drug pairs (bepridil + sertraline) and (sertraline + toremifene), with each drug dosed to yield ~25% survival, yield a greater than additive effect (i.e., greater than 50% survival). Following that, one of the pairs may warrant a test in an NHP model of EVD. This is currently advised, as drugs that have shown activity in rodent models as single agents have often failed in the NHP model [69]. Such an approach, when coupled with serial measurements of viral load could also provide initial estimates of the in vivo IC_50_ in a mammal, which could then be leveraged to more accurately simulate clinical trials in humans, allowing more precise selection of combination agents, dose selection and dosing interval.

In recent years, other combinations [23,24,26] (also see Supplemental Table S8) and other approved drugs have been identified with anti-EBOV activity in cell cultures; the latter include tilorone, pyronaridine and quinacrine [70], teicoplanin [71] and arbidol [72]. Ones deemed suitable for oral delivery may warrant additional studies as drug combinations. In addition, if approved, several investigational drugs may warrant testing in combinations. These include novel selective estrogen receptor modulators [73] and amodiaquine analogues [74], agents that target the HR2 region of EBOV GP2 [75,76], potentially broad-spectrum drugs that target the viral polymerase [77], or drugs that target other EBOV or host cell proteins or their interactions [78,79,80]. Moreover, the possibility exists of adding a third drug as supported by current therapeutic strategies against HIV [19,20] and HCV [21]. In the case of EBOV, such combinations could substantially increase potency, particularly if the agents work via different mechanisms leading to multiplicative rather than additive antiviral effects. Furthermore, as many drugs, such as bepridil, sertraline and toremifene [6,7,47] show similar activity against multiple strains of Ebolavirus as well as against Marburgvirus, the potential exists to identify a drug combination that will inhibit most, if not all, filoviruses.

## Figures and Tables

**Table 1:** Highly synergistic drug pairs from Ref. [22], rank ordered based on Matrix 3, Log Volume scores.

Drug 1	Drug 2	Matrix 1 (DBSumNeg)	Matrix 2 (DBSumNeg)	Matrix 3 (DBSumNeg)	Matrix 3 (Log Volume ^1^)
Aripiprazole	Piperacetazine	nd	nd	−5.14	28.0
Bepridil	Sertraline	nd	nd	−3.06	17.2
Sertraline	Clomiphene	−5.47	−5.85	−2.45	16.2
Sertraline	Toremifene	−4.40	−4.75	−3.09	16.2
Apilimod	Clomiphene	−4.90	na ^2^	−1.79	10.2
Apilimod	Azithromycin	nd	nd	−2.35	9.4
Apilimod	Toremifene	−4.35	na ^2^	−0.59	5.2

Drugs were tested against EBOV/Mak in Huh7 cells (moi 0.21) in three 6 × 6 checkerboard experiments: Matrix 1, Matrix 2, and Matrix 3 [22] (all data are available in Datasets 2 and 3, which accompany Ref. [22] as well as at: https://tripod.nih.gov/matrix-client/ (accessed on 1 March 2021). DBSumNeg is the sum of deviations from the Bliss model, a measure of synergy. Data from the Matrix 3 test were also analyzed with MacSynergy, which considers scores >9, Strong; >5, moderate; 2–5, minor; <2 insignificant. ^1^ Log Volume scores are at the 99.9% confidence level. ^2^ The apilimod used in this experiment did not have the correct chemical structure [22,46]. na, not applicable; nd, not done.

**Table 2:** Rationale for choice of top oral doses.

Drug	IP Dose (mg/kg)	Regimen	Survival (%)	Oral Dose (mg/kg)	Oral Vehicle	Oral C_max_ (μM)	Top Oral Dose (mg/kg)	Oral LD_50_ (mg/kg)
Bepridil	12	BID	100	150	Acacia	1.5	500	2069
Sertraline	10	BID	70	10	Saline	0.43	60	336
Toremifene	60	QOD	50	10	Saline	0.16	200	3000

Mouse survival data are from references [6,7]. Bepridil C_max_ is from reference [35]. C_max_ for sertraline and toremifene were determined for this study. BID, twice daily dosing (d0–d9); QOD, dosing d0, d1, 3, 5, 7, and 9. Oral LD_50_ (lethal dose for 50% of animals tested) data are from PubChem (https://pubchem.ncbi.nlm.nih.gov/) (accessed 21 January 2021). Bepridil and sertraline lethal dose values are for mice, and toremifene for rats. The sertraline value was reported as LDLo (Lethal dose low, the lowest dose to have caused death).

**Table 3:** Oral PK in mice following drug administration in V8.

Drug	Dose (mg/kg)	Cmax (ng/mL)	Cmax (μM)	MRT (h)	T_1/2_ (h)	IC_50_^1^ (μM)	C_max_/ IC_50_^1^
Bepridil	150	765	2.09	6	0.5	4.5	0.46
	500	1948	5.31	9	0.5		1.18
Sertraline	30	188	0.61	11	1.0	2.6	0.23
	60	453	1.48	5	1.0		0.56
Toremifene	100	498	1.23	6	0.5	1.8	0.67
	200	1295	3.19	16	0.2		1.74

^1^ average of IC_50_s from two studies (see Supplementary Table S5). MRT, Mean Residence Time.

**Table 4:** In vitro PD parameters for drug combinations.

	Sertraline–Bepridil		Sertraline–Toremifene	
Parameters	Drug 1 Sertraline	Drug 2 Bepridil	Drug 1 Sertraline	Drug 2 Toremifene
*m_i_*	2.736	4.043	1.996	1.628
*IC*_50,*i*_	2.961	2.688	1.888	0.7673
a	1.005		1.013	
R^2^	0.9435		0.9317	

$IC_{50,i}$ and $m_{i}$ are the concentration of drug *i* (μM) required for 50% viral inhibition and the hill coefficient of drug *i* when drugs are applied in combination. a is a power factor, capturing the possible synergistic effects of drug combinations. R^2^ is a statistical quantity indicating the proportion of variance explained by a model.

## Data Availability

Data are available upon request from a corresponding author (J.M.W., J.T.S.).

## Supplementary Materials

The following are available online at https://www.mdpi.com/2076-2607/9/3/566/s1, Figure S1: Pharmacokinetic model of bepridil, Figure S2: Pharmacokinetic model of sertraline, Figure S3: Pharmacokinetic model of toremifene, Figure S4: Pharmacodynamic projections of single drugs’ efficacies assuming various in vivo IC_50_s, Figure S5: Apilimod does not protect mice challenged with ma-EBOV, Table S1: PK parameters for bepridil, Table S2: PK parameters for sertraline, Table S3: PK parameters for toremifene, Table S4: In vitro PD parameters for single drugs, Table S5: Predicted drug exposures (C_max_) in humans following PO administration compared to drug efficacy (IC_50_) vs. EBOV in cultured liver cells, Table S6: Prior studies of drug efficacies as single agents in mouse models of lethal EBOV infection, Table S7: Solubility of bepridil, sertraline and toremifene in ten test vehicles, Table S8: Additional in vitro drug synergy tests, and a Supplementary Document.
