# Supplementary material for: Formulation, Stability, Pharmacokinetic, and Modeling Studies for Tests of Synergistic Combinations of Orally Available Approved Drugs against Ebola Virus In Vivo
Source: Microorganisms. 2021 Mar 10;9(3):566. doi: 10.3390/microorganisms9030566 (PMC7998926; doi:10.3390/microorganisms9030566)
Supplement: Supplementary file 1 [file microorganisms-09-00566-s001.zip › microorganisms-1123604-S/FinchSuppDocs0308/Finch.SD.docx]

Finch et al. 2021

**Supplementary Document**

*Preparation of Vehicles for Formulation Tests*

Vehicle #1 (Aqueous Suspension Vehicle, ASV) was prepared as follows: Sodium chloride (9 mg/ml final concentration) and sterile water for injection (70% final volume) were stirred for 5 min until the sodium chloride was dissolved. Benzyl alcohol (0.9% final concentration, v/v) was added, and the mixture was stirred for 5 min until it was a clear solution. Tween 80 (0.4% final concentration, v/v) was then added, and the mixture was again stirred for 10 min until Tween 80 dissolved. Carboxymethylcellulose (5 mg/ml final concentration) was then added while stirring. The solution mixture was stirred for 5 min before water was added Q.S. (as much as needed) to bring to the final volume. The ASV was stirred for 5 min, then autoclaved for 30 min at 121°C, and allowed to cool to room temperature for 90 min. The ASV was stored refrigerated until use.

Vehicle #2 (20% Captisol) was prepared by mixing Captisol (20% w/v) with water (~70% of final volume) using a magnetic stirrer for 10 min until all solid was dissolved, then water was added Q.S. to the final volume and the solution was mixed for an additional 5 min.

Vehicles #3 and 6 were prepared by mixing the listed individual vehicle components at the appropriate concentration on a magnetic stirrer for 5 to 10 min. Solutol was melted at 50–60°C before use. 0.1% Tween 20 in water was prepared by mixing Tween 20 (0.1% v/v) with water (99.9% v/v) on a magnetic stirrer for 10 min.

Formulations in Vehicle #1, 2, 3 and 6 were prepared by mixing the appropriate amount of test article in the vehicle to achieve the target concentration. The formulations were mixed using a vortex mixer for 0 to 1 min and sonication for 15 to 30 min, and then stirred on a magnetic stirrer overnight.

Formulations in Vehicle #4 (Vegetable oil) were prepared by mixing the appropriate amount of test article) in Ethanol (100% of final volume) using a vortex mixer for 1 min and sonication for 20 to 30 min, then an equal volume of vegetable oil was added, then the mixture was mixed using a vortex mixer for 2 min. The mixture was placed under a speedvac (a centrifuge with vacuum pump). The level of ethanol was checked every 30 min and the mixture was mixed using a vortex mixer for 30 sec at that same time; the speedvac process continued for 1 hr until all of the ethanol was removed. This procedure produced a formulation of test article in vegetable oil at the desired concentration.

Formulations in Vehicle #5 (PEG 400) were prepared by mixing the appropriate amount of test article in PEG 400 using a vortex mixer for 1 min and sonication for 15 min, then stirring in a water bath at 40°C for overnight.

Formulations in Vehicle #7–10 were prepared by step-wise addition of each vehicle component, in the order listed, to the appropriate amount of test article to achieve the target concentration. The formulations were mixed using a vortex mixer for 1 min and sonication for 15 to 30 min between the addition of each vehicle component until a clear solution or homogeneous suspension was obtained.

*Preparation of Bepridil, Sertraline and Toremifene for Stability, Efficacy, Pharmacokinetic, and Tolerability Studies in Vehicle 8*

Bepridil, sertraline and toremifene were prepared in Vehicle 8 [80% PEG 400/20% of 0.1% Tween 20 in Water] as follows:

1. Prepare 0.1% Tween 20 in Water

- 1. Measure 49.95 mL sterile water into a bottle
  2. Place water on magnetic stir plate,
  3. Place magnetic stirrer in water and turn on the stirrer.
  4. Measure out 0.05 mL Tween 20 and add to the water while stirring.
  5. Stir for 10 min
  6. Solution should be clear. Set aside at room temperature and continue to step 2.

1. Weigh out test articles (sertraline, bepridil and toremifene) into clear vials.
2. Add 0.8 mL PEG 400 to each tube of test article. Cap the vials.
   1. Bepridil: Vortex for 1 min then sonicate for 15 min
      1. Solution will be clear and slightly orange after this step
   2. Sertraline: Vortex for 1 min then sonicate for 15 min
      1. Solution will be suspension after this step
   3. Toremifene: Vortex for 1 min then sonicate for 15 min
      1. Solution will be a white suspension after this step
3. To the test article + PEG 400, add 0.2 mL of the 0.1% Tween 20 in water prepared in Step 1.
   1. Bepridil: Vortex 1 min
      1. Solution should be clear orange after this step
   2. Sertraline: Vortex for 1 min, then sonicate for 15 min
      1. Solution should be clear and colorless after this step
   3. Toremifene: Vortex for 1 min, then sonicate for 30 min
      1. Solution should be clear and colorless after this step

*Special Notes on Preparation of bepridil, sertraline and toremifene in Vehicle 8*

Unless stated, preparations of bepridil, sertraline and toremifene in Vehicle 8 were always prepared fresh on day 0 (d0) of a study and then stored at 4°C. Sertraline and toremifene were always usable directly from the refrigerator (i.e. no precipitates, not solid). In contrast, when removed from the refrigerator, bepridil was always solid (as if it had frozen) and contained precipitate. Three full cycles of the following three steps were always performed to resolubilize bepridil:

1. Incubate 5 min at 37°C
2. Sonicate for 15 min
3. Vortex 1min

Sonication was done using a bath sonicator (Branson #2510), which has no specific energy settings. No heat was applied during drug preparation. Once resolubilized, bepridil should be used as soon as possible. If delayed and the bepridil re-solidifies, repeat the above three steps (2 or 3 full cycles) to get bepridil back into solution.

*Sources of Drugs for Cell-Based Infection Tests*

| **Name** | **Supplier** | **Cat. #** |
| --- | --- | --- |
| Amodiaquine | Sigma | A2799-5G |
| Apilimod | Axon Medchem | 1369 |
| Aripiprazole | Selleck | S1975 |
| Bepridil | Sigma | B5016-100MG |
| Favipiravir | Selleck | S7975 |
| Piperacetazine | USP | na |
| Sertraline | Toronto Research Chemicals | S280000 |
| Toremifene | Selleck | S1176 |

*Sources of Drugs for Formulation Tests*

| **Name** | **Supplier** | **Cat. #** |
| --- | --- | --- |
| Bepridil Hydrochloride | Sigma-Aldrich | B5016 |
| Clomiphene Citrate Salt | Sigma-Aldrich | C6272 |
| Toremifene Citrate | Carbosynth | FT28330 |
| Sertraline Hydrochloride | Toronto Research Chemicals | S280000 |

*Sources of Vehicle Components for Formulation Tests*

| **Name** | **Supplier** | **Cat. #** |
| --- | --- | --- |
| Benzyl alcohol | Sigma-Aldrich | 305197 |
| Capmul MCM NF | ABITEC Corporation | N/A |
| Captisol | Captisol | N/A |
| Carboxymethylcellulose Sodium, High Viscosity | Spectrum Chemicals | CA194 |
| Ethanol | Spectrum Chemicals | EI068 |
| NMP (1-Methyl-2-pyrrolidinone) | Sigma-Aldrich | 328634 |
| PEG 300 | Spectrum Chemicals | PO108 |
| PEG 400 | Spectrum Chemicals | PO138 |
| Polysorbate 20 (Tween 20) | Spectrum Chemicals | PO132 |
| Polysorbate 80 (Tween 80) | Spectrum Chemicals | PO138 |
| Propylene Glycol (PG) | Spectrum Chemicals | PR130 |
| Sodium Chloride | Spectrum Chemicals | SO155 |
| Sterile Water for Injection | Henry Schein Vet | 002488 |
| Solutol (Kolliphore HS 15) | Sigma-Aldrich | 42966 |
| Vegetable Oil (Crisco Pure Vegetable Oil) | The J.M. Smucker Company | N/A |
