# Supplementary material for: Formulation, Stability, Pharmacokinetic, and Modeling Studies for Tests of Synergistic Combinations of Orally Available Approved Drugs against Ebola Virus In Vivo
Source: Microorganisms. 2021 Mar 10;9(3):566. doi: 10.3390/microorganisms9030566 (PMC7998926; doi:10.3390/microorganisms9030566)
Supplement: Supplementary file 1 [file microorganisms-09-00566-s001.zip › microorganisms-1123604-S/FinchSuppDocs0308/Finch.SFs.rr.docx]

Formulation, Stability, Pharmacokinetic and Modeling Studies for Tests of Synergistic Combinations of Orally Available Approved Drugs Against Ebola Virus In Vivo

Finch et al. 2021

Supplemental Figures


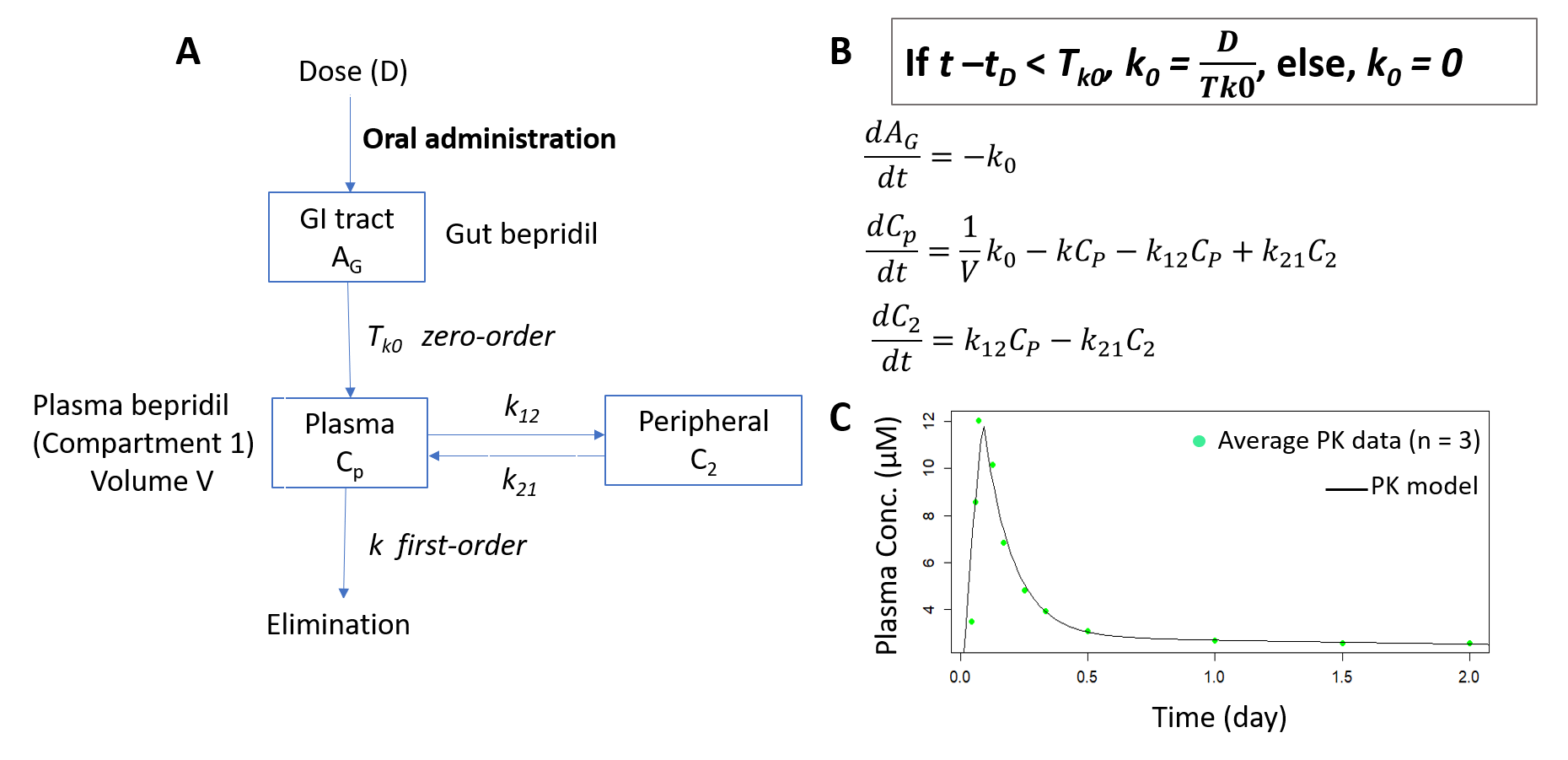


**Supplemental Figure 1. Pharmacokinetic model of bepridil**. (**A)** Schematic PK model of bepridil. Dose, *D*, of bepridil is orally administered into GI tract (*A_G_*), and it is then absorbed into the Plasma compartment (*C_P_*, with volume *V*) following a zero-order absorption. Bepridil in plasma compartment (*C_P_*) moves to the Peripheral compartment (*C_2_*) at rate *k_12_* and reversely at rate *k_21_*. Bepridil is eliminated at rate *k* from the Plasma compartment (*C_P_*). (**B)** Differential equations describing the pharmacokinetics of bepridil in each compartment. (**C)** Projections of plasma bepridil levels and the average concentrations of bepridil measured in the plasma of volunteers following oral administration of 400 mg bepridil-HCl. (See Reference [35] in the Main Text.)


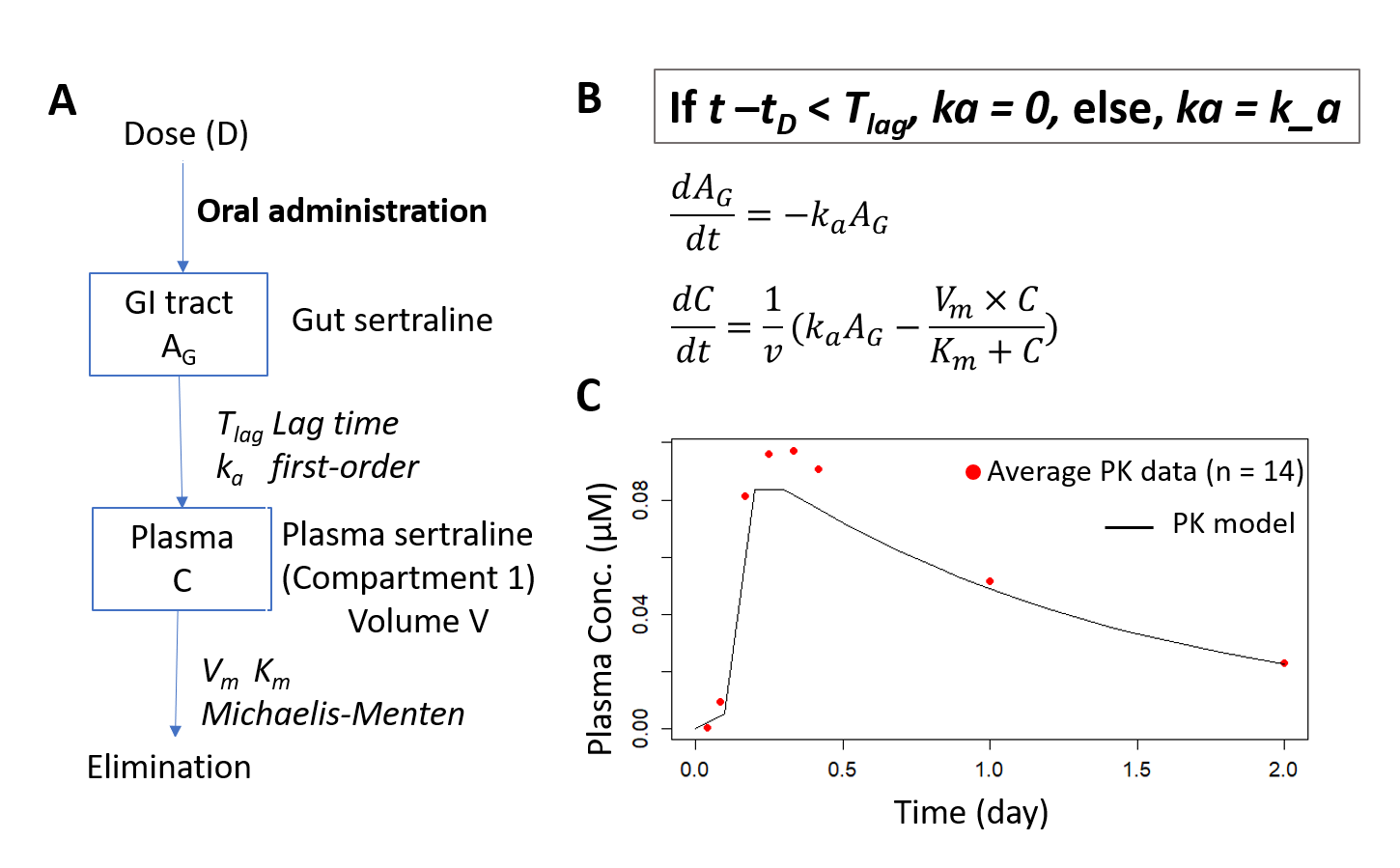


**Supplemental Figure 2. Pharmacokinetic model of sertraline**. (**A)** Schematic PK model of sertraline. Dose, *D*, of sertraline is orally administered into GI tract (*A_G_*), and it is then absorbed into the Plasma compartment (*C*, with volume *V*) with delay, *T_lag_*, at rate *k_a_*. Sertraline is eliminated from the Plasma compartment (*C*) following Michaelis Menten reaction with *V_m_* and *K_m_*. (**B)** Differential equations describing the PK of sertraline in each compartment. (**C)** Projections of plasma sertraline levels and the average concentrations of sertraline measured in the plasma of volunteers following oral administration of 100 mg of sertraline-HCl. (See Reference [37] in the Main Text.)


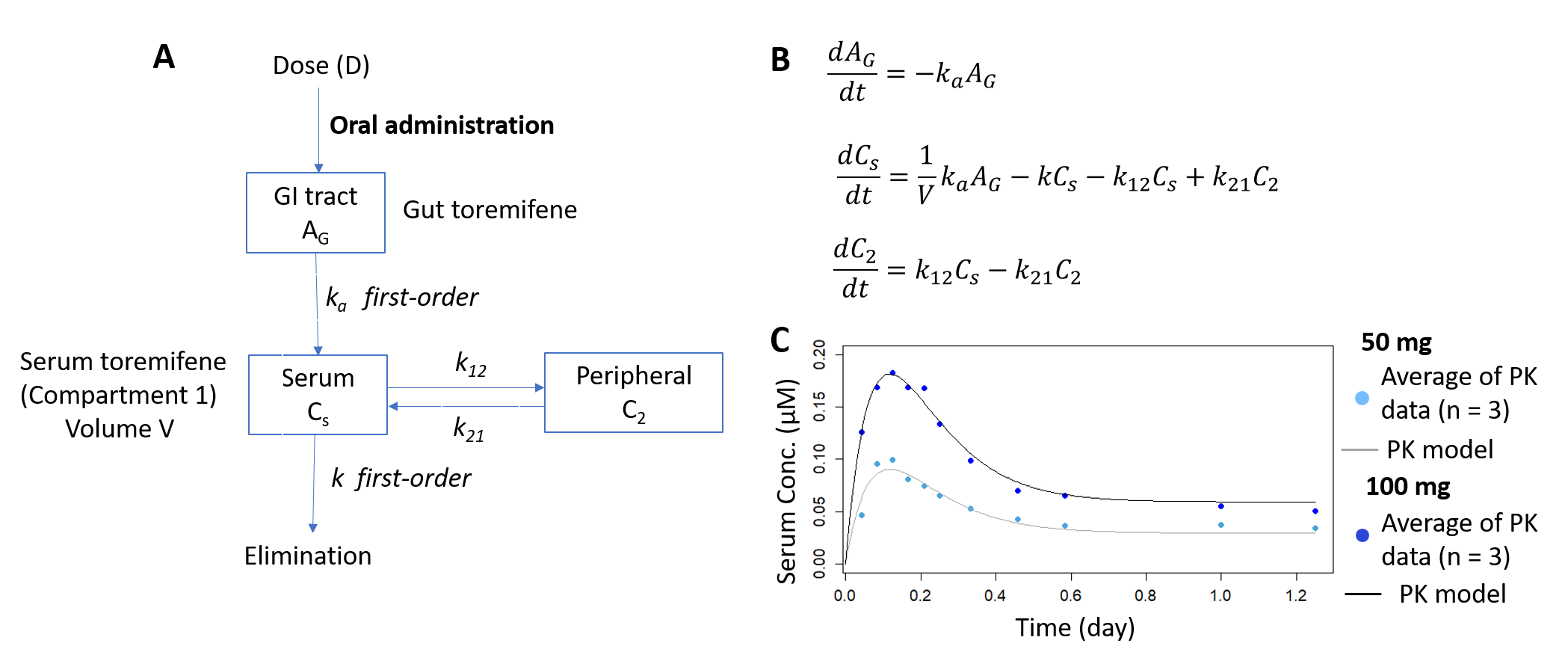


**Supplemental Figure 3. Pharmacokinetic model of toremifene**. (**A)** Schematic PK model of toremifene. Dose, *D*, of toremifene is orally administered into GI tract (*A_G_*), and it is then absorbed into the Serum compartment (*C_s_*, with volume *V*) at rate *k_a_*. Toremifene in serum compartment (*C_s_*) moves to the Peripheral compartment (*C_2_*) at rate *k_12_* and reversely at rate *k_21_*. Toremifene is eliminated at rate *k* from the Serum compartment (*C_s_*). (**B)** Differential equations describing the PK of toremifene in each compartment. (**C)** Projections of serum toremifene levels and the average concentrations of toremifene measured in the serum of volunteers following oral administration of 50 or 100 mg of toremifene as indicated in Panel C. (See Reference [36] in the Main Text.)

**
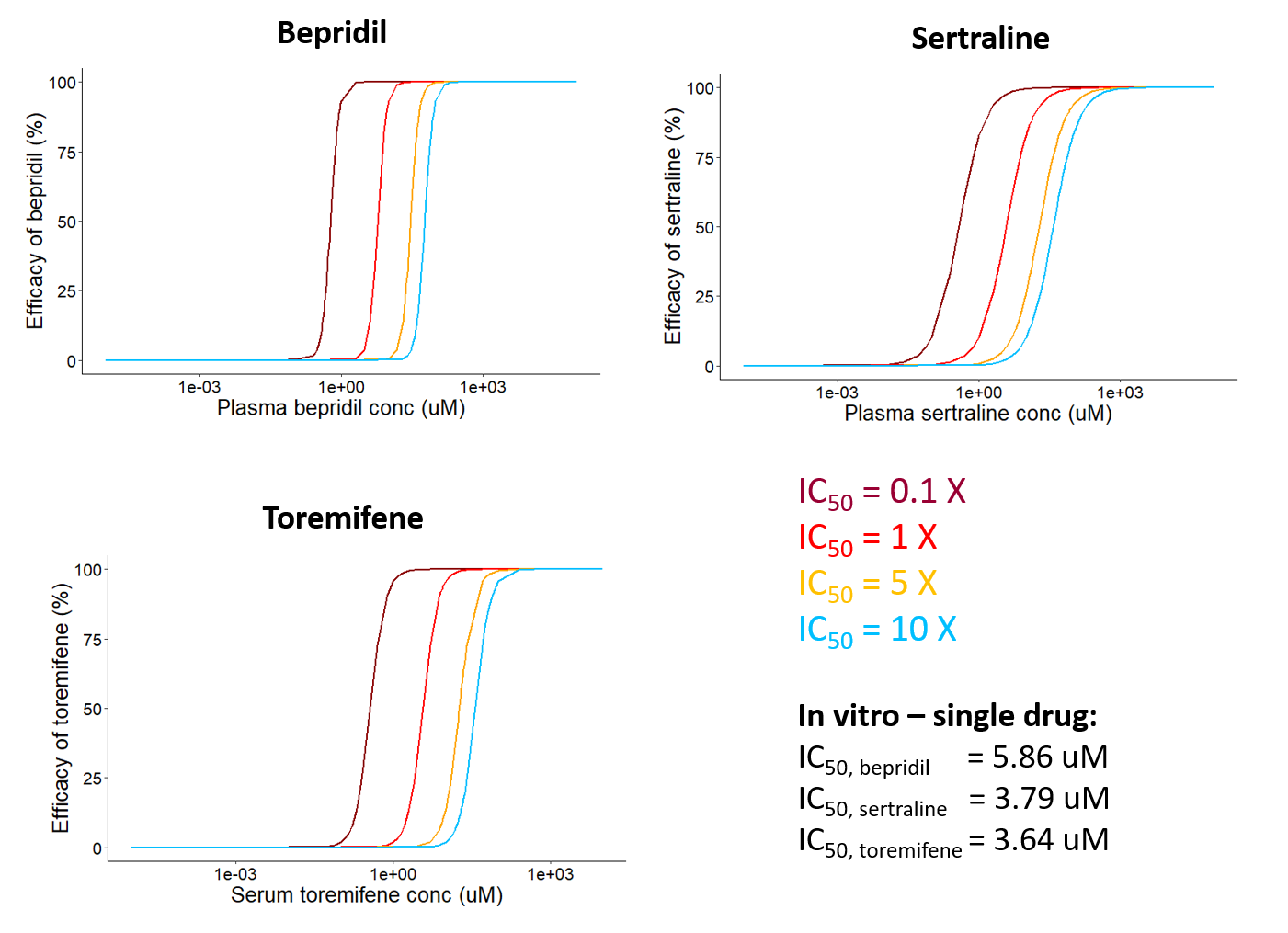
Supplemental Figure 4. Pharmacodynamic projections of the efficacies of single drugs assuming various in vivo IC50s.** Line colors indicate the assumptions of an in vivo IC50s as follows: sky blue for 10X, orange for 5X, red for 1X and dark red for 0.1X of in vitro IC_50_. Antiviral efficacy of a single drug changes according to its drug concentration.


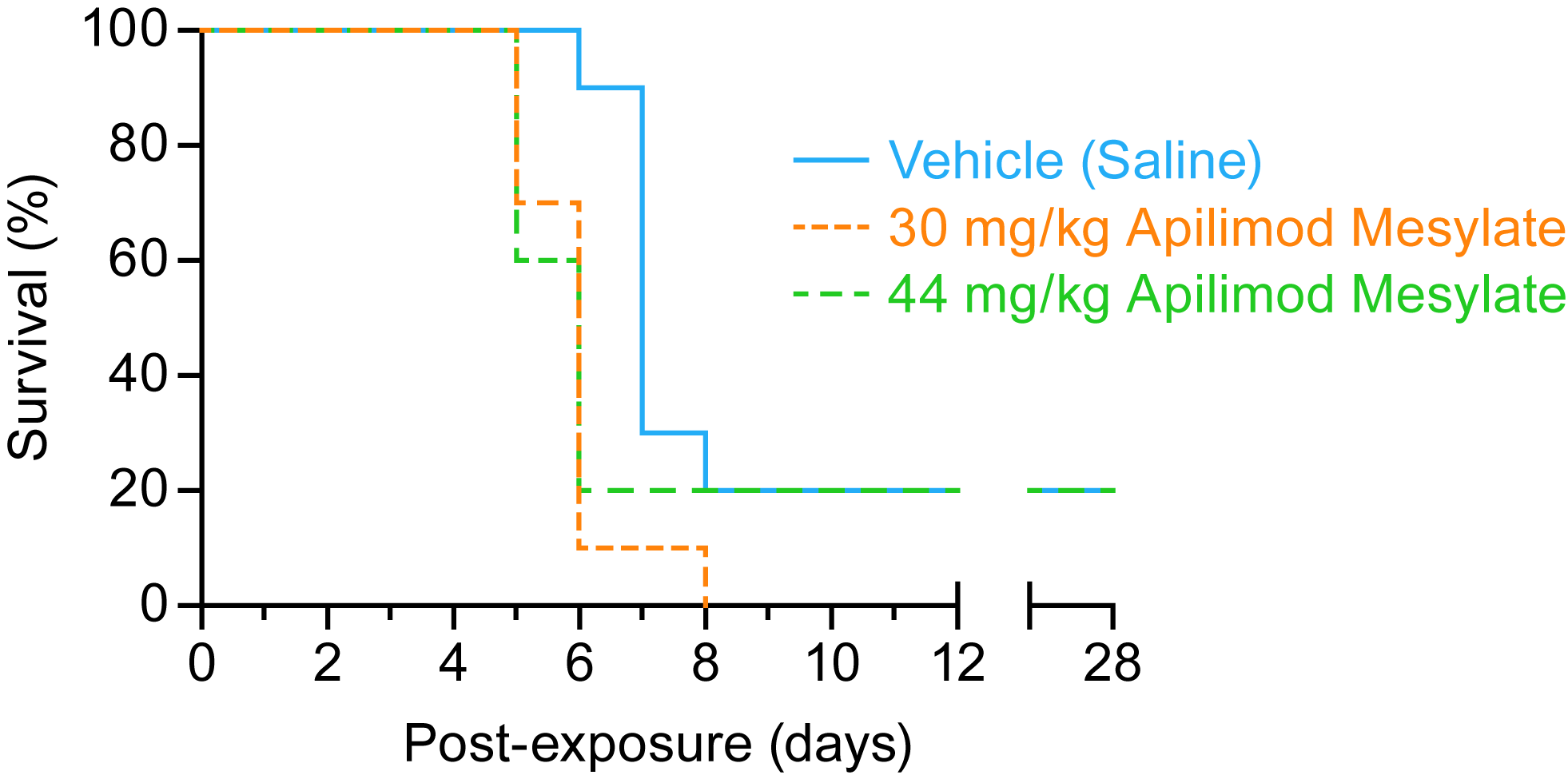


**Supplementary Figure 5**. **Apilimod does not protect mice challenged with mouse adapted EBOV (ma-EBOV)**. Female C56BL/6 mice (10/group) were treated with the indicated dose of apilimod (in 0.9% saline) or vehicle control. ~4 hr later they were infected with 246 PFU of ma-EBOV. They were then treated daily with the same dose of apilimod, prepared freshly from a pre-weighed and stored packet (or vehicle), for an additional 9 days. The graph shows daily survival rates. See the Methods section for details. A PK study in mice revealed that a single dose of 10 mg/kg apilimod administered IP resulted in a C_max_ of 2.53 µM, which is well above the in vitro IC50 against EBOV in Huh7 cells (Supplemental Table 5).
