# Supplementary material for: Formulation, Stability, Pharmacokinetic, and Modeling Studies for Tests of Synergistic Combinations of Orally Available Approved Drugs against Ebola Virus In Vivo
Source: Microorganisms. 2021 Mar 10;9(3):566. doi: 10.3390/microorganisms9030566 (PMC7998926; doi:10.3390/microorganisms9030566)
Supplement: Supplementary file 1 [file microorganisms-09-00566-s001.zip › microorganisms-1123604-S/FinchSuppDocs0308/Finch.STs.031021.docx]

Formulation, Stability, Pharmacokinetic and Modeling Studies for Tests of Synergistic Combinations of Orally Available Approved Drugs Against Ebola Virus In Vivo

Finch et al. 2021

Supplemental Tables (see end of document for References to Supplemental Tables)

| ***T_k0_*** (day) | 0.0873 |
| --- | --- |
| ***V*** | 25.2 |
| ***k_12_*** (per day) | 6.71 |
| ***k_21_*** (per day) | 1.61 |
| ***k*** (per day) | 0.348 |

**Supplemental Table 1. PK parameters for bepridil**

| ***T_lag_*** (day) | 0.0976 |
| --- | --- |
| ***k_a_*** (per day) | 25.3 |
| ***V*** | 909 |
| ***v_m_*** (per day) | 2.61×10^4^ |
| ***k_m_*** (per day) | 32.2 |

**Supplemental Table 2. PK parameters for sertraline**

| ***k_a_*** (per day) | 16.6 |
| --- | --- |
| ***V*** | 354 |
| ***k_12_*** (per day) | 4.28 |
| ***k_21_*** (per day) | 0.992 |
| ***k*** (per day) | 0.000198 |

**Supplemental Table 3. PK parameters for toremifene**

| **Drug** | **IC_50_ (µM)** | **Hill coefficient** |
| --- | --- | --- |
| **Bepridil** | 5.86 | 4.859375 |
| **Sertraline** | 3.79 | 1.628 |
| **Toremifene** | 3.64 | 3.057 |

**Supplemental Table 4. In vitro PD parameters for single drugs.** Data are from Ref. [1]

| **Drug** | **IC_50_**  **(Huh7)**  **(μM)** | **IC_50_**  **(HepG2)**  **(μM)** | **IC_50_**  **(average)**  **(μM)** | **Oral Dose ^1^**  **(mg)** | **C_max_**  **(PO)**  **(μM)** | **C_max_/**  **IC_50_** | **Reference**  **PK Data** |
| --- | --- | --- | --- | --- | --- | --- | --- |
| apilimod | 0.17 | nd | 0.17 | 70 | 0.26 | 1.56 | [2] |
| aripiprazole | 7.8 | 3.76 | 5.78 | 30 | 1.01 ^2^ | 0.17 | [3] |
| azithromycin | 10.75 | nd | 10.75 | 500 | 0.64 | 0.06 | FDA |
| bepridil | 5.86 | 3.21 | 4.53 | 400 | 2.43 | 0.54 | [4] |
| clomiphene | 1.96 | 0.76 | 1.36 | 50 | 0.05 | 0.04 | [5] |
| piperacetazine | 7.58 | 3.30 | 5.44 | na | na | na | na |
| sertraline | 3.79 | 1.44 | 2.62 | 200 | 0.54 ^3^ | 0.21 | [6] |
| toremifene | 3.64 | 0.03 | 1.83 | 60 | 1.97 ^4^ | 1.08 | [7] |

**Supplemental Table 5. Predicted drug exposures (C_max_) in humans following PO administration compared to drug efficacy (IC_50_) against EBOV in cultured liver cells**. Values in column 2 are from Dyall ([1]; Dataset 1, all at moi 0.21; all from Sheet 4 of the Excel workbook except clomiphene and sertraline, which are from Sheet 2 of the Excel workbook). Values in column 3 are from Table 1 in Johansen [8]). Values in column 4 are the averages of the values in columns 2 and 3. ^1^C_max_ after a single oral dose, unless specified. ^2^C_max_ after 14 days of daily dosing. ^3^C_max_ after 30 days of daily dosing to females (18-45 years of age); the 200 mg dose was reached after 3 days of dose increases starting at 50 mg. ^4^C_max_ after multiple days of daily dosing to healthy adults (see Figure 6 in [7]). FDA refers to the FDA package insert for azithromycin. Abbreviations: na, not available (piperacetazine is only authorized for veterinary use in the USA); nd, not done; PO, Per Os (by mouth).

| **Drug** | **Dose**  **(mg/kg)** | **Regimen** | **Virus** | **Survival**  **(%)** | **Reference** |
| --- | --- | --- | --- | --- | --- |
| Apilimod | 44 | IP, SID | EBOV | 0 | * |
| Aripiprazole | 20 | IP, SID | EBOV | 10 ^1^ | [8] |
| Azithromycin | 100 | IP, SID | EBOV | 10, 30, 60 ^2^ | [9] |
| Bepridil | 12 | IP, BID | EBOV | 100 ^3^ | [8] |
|  | 12 | IP, BID | MARV | 90 ^4^ | [10] |
| Clomiphene | 60 | IP, QOD | EBOV | 90 ^5^ | [11] |
|  | 60 | IP, BID | EBOV | 10 ^6^ | [9] |
| Piperacetazine | nd | nd | nd | nd | nd |
| Sertraline | 10 | IP, BID | EBOV | 70 | [8] |
| Toremifene | 60 | IP, QOD | EBOV | 50 | [11] |

**Supplementary Table 6. Prior studies of drug efficacies as single agents in mouse models of lethal EBOV infection**. For the studies described in *(Supplementary Figure 5, this study) and in References 8, 10 and 11, mice were treated on day 0 (d0) and then on d1-9 as indicated (either SID, BID, or QOD) and observed for a total of 28 days. For the study described in Reference 9, mice were treated on d0 and then on d1-7 and observed for a total of 14 days. ^1^Mice became extremely somnolent, thwarting eating and drinking, which likely contributed to low survival. ^2^20% survival was seen in the control group in the study yielding 60% survival with azithromycin. ^3^90-100% survival has been seen dosing with 12 mg/kg, IP, SID (unpublished data). ^4^In the same report, 80% mice were protected if treated with 12 mg/kg bepridil, IP, SID. ^5^In the same report, a study comparing male and female mice (with fewer mice per group) yielded 60% and 40% protection of female and male mice, respectively (dosed with 12 mg/kg, IP, BID). ^6^In the same report, no mice were protected if treated with 21 mg/kg clomiphene, PO, SID. Abbreviations: BID, twice daily dosing; EBOV, Ebola virus; IP, intraperitoneal; MARV, Marburgvirus; nd, not done; QOD, dosing on d0, 1, 3, 5, 7, 9. SID, once daily dosing.

| **Vehicle #** | **Bepridil**  **(solubility)** | **Sertraline**  **(solubility)** | **Toremifene**  **(solubility)** |
| --- | --- | --- | --- |
| 1 | Suspension | Suspension | Suspension |
| 2 | Slightly hazy | Clear solution | Clear solution |
| 3 | Clear solution | Clear solution | Suspension |
| 4 | Clear solution | Clear solution | Non-homogeneous |
| 5 | Clear solution | Clear solution | Suspension |
| 6 | Clear solution | Clear solution | Suspension |
| 7 | Clear solution | Clear solution | Clear solution |
| 8 | Clear solution | Clear solution | Clear solution |
| 9 | Clear solution | Clear solution | Clear solution |
| 10 | Clear solution | Clear solution | Insoluble |

**Supplementary Table 7. Solubility of bepridil, sertraline and toremifene in ten test vehicles.** The vehicle formulations were: #1, Aqueous Suspension Vehicle; #2, 20% Captisol in water; #3, 37.5% PEG 400/ 37.5% Tween 20/25% Capmul MCM NF; #4, Vegetable Oil; #5, PEG 400; #6, 10% Solutol/90% PEG 400; #7, 5% NMP/95% PEG 300; #8, 80% PEG 400/ 20% of 0.1% Tween-20 in Water; #9, 3% NMP/45% PEG300/12% ethanol/40% sterile water; #10, 40% Propylene Glycol/30% Solutol HS 15/30% Sterile Water. Drug concentrations were: Bepridil, 25 mg/ml; Sertraline, 3 mg/ml; toremifene, 8.4 mg/ml. See the Supplementary Document for details on sample preparation.

| **Drug 1** | **Drug 2** | **MacSynery**  **(Av. LogV)** | **n** |
| --- | --- | --- | --- |
| Aripiprazole | Piperacetazine | 15.0 | 2 ^1^ |
| Aripiprazole | Amodiaquine | 3.2 | 3 |
| Aripiprazole | Bepridil | 7.5 | 2 |
| Favipiravir | Bepridil | 0.0 | 3 |
| Favipiravir | Amodiaquine | 7.6 | 3 |
| Favipiravir | Aripiprazole | 2.0 | 3 |
| Favipiravir | Ribavarin | 2.8 | 3 |
| Favipiravir | Sertraline | 0.9 | 2 |
| Favipiravir | Toremifene | 0.1 | 2 |
| Favipiravir | Azithromycin | 1.4 | 2 |
| Favipiravir | Clomiphene | 1.3 | 2 |
| Favipiravir | Favipiravir | 0.0 | 2 |

**Supplementary Table 8. Additional *in vitro* drug synergy tests.** All synergy tests were performed in Huh7 cells with Ebov/Mak at moi 0.21 and analyzed using MacSynergy software as described in [1]. Data are presented at the 99.9% confidence level. ^1^In a third test using higher top doses of both drugs, the LogV was 71.95, which may be erroneous (https://www.uab.edu/images/pediatrics/ID/MacSynergy.pdf). Cytotoxicity was analyzed in parallel in three plates in all experiments as described in the Methods section. For the pairs showing strong (aripiprazole + piperacetazine) or moderate ((favipiravir + amodiaquine); (aripiprazole + bepridil)) synergy, toxicity was generally < 5-20% throughout the plate, with higher toxicity seen in some wells containing high concentrations of drugs. Abbreviations: Av. LogV, average log volume; n, number of replicate experiments; each experiment performed in triplicate plates.

References to Supplemental Tables, Finch et al, 2021

1. Dyall, J.; Nelson, E. A.; DeWald, L. E.; Guha, R.; Hart, B. J.; Zhou, H.; Postnikova, E.; Logue, J.; Vargas, W. M.; Gross, R.; Michelotti, J.; Deiuliis, N.; Bennett, R. S.; Crozier, I.; Holbrook, M. R.; Morris, P. J.; Klumpp-Thomas, C.; McKnight, C.; Mierzwa, T.; Shinn, P.; Glass, P. J.; Johansen, L. M.; Jahrling, P. B.; Hensley, L. E.; Olinger, G. G.; Thomas, C.; White, J. M. Identification of combinations of approved drugs with synergistic activity against ebola virus in cell cultures. *J. Infect. Dis.* **2018**, *218*, S672–S678.

2. Wada, Y.; Cardinale, I.; Khatcherian, A.; Chu, J.; Kantor, A. B.; Gottlieb, A. B.; Tatsuta, N.; Jacobson, E.; Barsoum, J.; Krueger, J. G. Apilimod inhibits the production of IL-12 and IL-23 and reduces dendritic cell infiltration in psoriasis. *PLoS One* **2012**, *7*, e35069.

3. Mallikaarjun, S.; Salazar, D. E.; Bramer, S. L. Pharmacokinetics, tolerability, and safety of aripiprazole following multiple oral dosing in normal healthy volunteers. *J. Clin. Pharmacol.* **2004**, *44*, 179–187.

4. Wu, W. N.; Pritchard, J. F.; Ng, K. T.; Hills, J. F.; Uetz, J. A.; Yorgey, K. A.; McKown, L. A.; O’Neill, P. J. Disposition of bepridil in laboratory animals and man. *Xenobiotica* **1992**, *22*, 153–169.

5. Ghobadi, C.; Mirhosseini, N.; Shiran, M. R.; Moghadamnia, A.; Lennard, M. S.; Ledger, W. L.; Rostami-Hodjegan, A. Single-dose pharmacokinetic study of clomiphene citrate isomers in anovular patients with polycystic ovary disease. *J. Clin. Pharmacol.* **2009**, *49*, 147–154.

6. Ronfeld, R. A.; Tremaine, L. M.; Wilner, K. D. Pharmacokinetics of sertraline and its N-demethyl metabolite in elderly and young male and female volunteers. *Clin Pharmacokinet* **1997**, *32 Suppl 1*, 22–30.

7. Anttila, M.; Valavaara, R.; Kivinen, S.; Mäenpää, J. Pharmacokinetics of toremifene. *J Steroid Biochem* **1990**, *36*, 249–252.

8. Johansen, L. M.; DeWald, L. E.; Shoemaker, C. J.; Hoffstrom, B. G.; Lear-Rooney, C. M.; Stossel, A.; Nelson, E.; Delos, S. E.; Simmons, J. A.; Grenier, J. M.; Pierce, L. T.; Pajouhesh, H.; Lehár, J.; Hensley, L. E.; Glass, P. J.; White, J. M.; Olinger, G. G. A screen of approved drugs and molecular probes identifies therapeutics with anti-Ebola virus activity. *Sci. Transl. Med.* **2015**, *7*, 290ra89.

9. Madrid, P. B.; Panchal, R. G.; Warren, T. K.; Shurtleff, A. C.; Endsley, A. N.; Green, C. E.; Kolokoltsov, A.; Davey, R.; Manger, I. D.; Gilfillan, L.; Bavari, S.; Tanga, M. J. Evaluation of ebola virus inhibitors for drug repurposing. *ACS Infect. Dis.* **2015**, *1*, 317–326.

10. DeWald, L. E.; Dyall, J.; Sword, J. M.; Torzewski, L.; Zhou, H.; Postnikova, E.; Kollins, E.; Alexander, I.; Gross, R.; Cong, Y.; Gerhardt, D. M.; Johnson, R. F.; Olinger, G. G.; Holbrook, M. R.; Hensley, L. E.; Jahrling, P. B. The calcium channel blocker bepridil demonstrates efficacy in the murine model of marburg virus disease. *J. Infect. Dis.* **2018**, *218*, S588–S591.

11. Johansen, L. M.; Brannan, J. M.; Delos, S. E.; Shoemaker, C. J.; Stossel, A.; Lear, C.; Hoffstrom, B. G.; Dewald, L. E.; Schornberg, K. L.; Scully, C.; Lehár, J.; Hensley, L. E.; White, J. M.; Olinger, G. G. FDA-approved selective estrogen receptor modulators inhibit Ebola virus infection. *Sci. Transl. Med.* **2013**, *5*, 190ra79.
