# Supplementary figures and images for: Formulation, Stability, Pharmacokinetic, and Modeling Studies for Tests of Synergistic Combinations of Orally Available Approved Drugs against Ebola Virus In Vivo
Source: Microorganisms. 2021 Mar 10;9(3):566. doi: 10.3390/microorganisms9030566 (PMC7998926; doi:10.3390/microorganisms9030566)

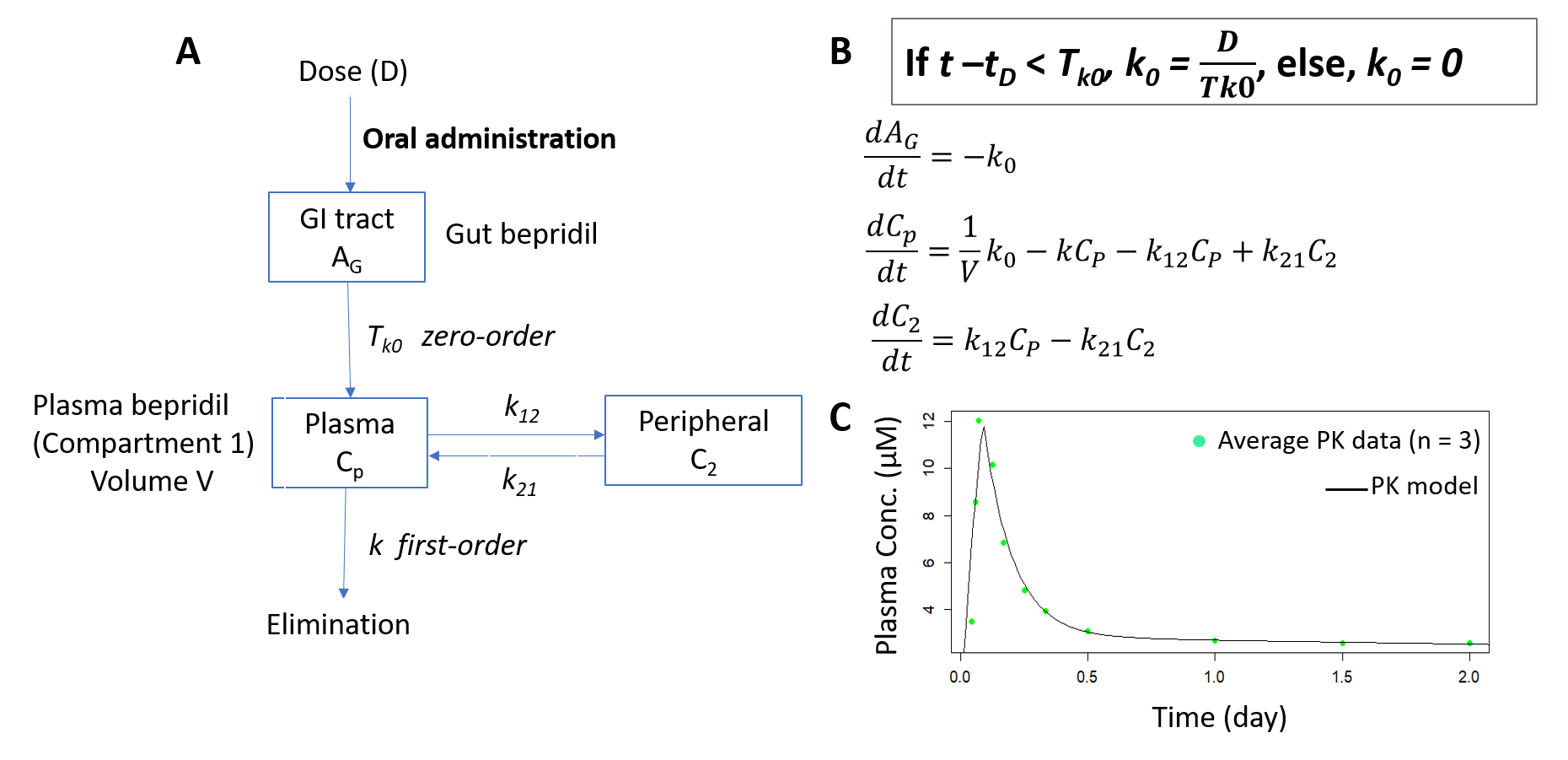

Supplement: Supplementary file 1 [file microorganisms-09-00566-s001.zip › microorganisms-1123604-S/Supplementary Figures/SupFig1.tif]

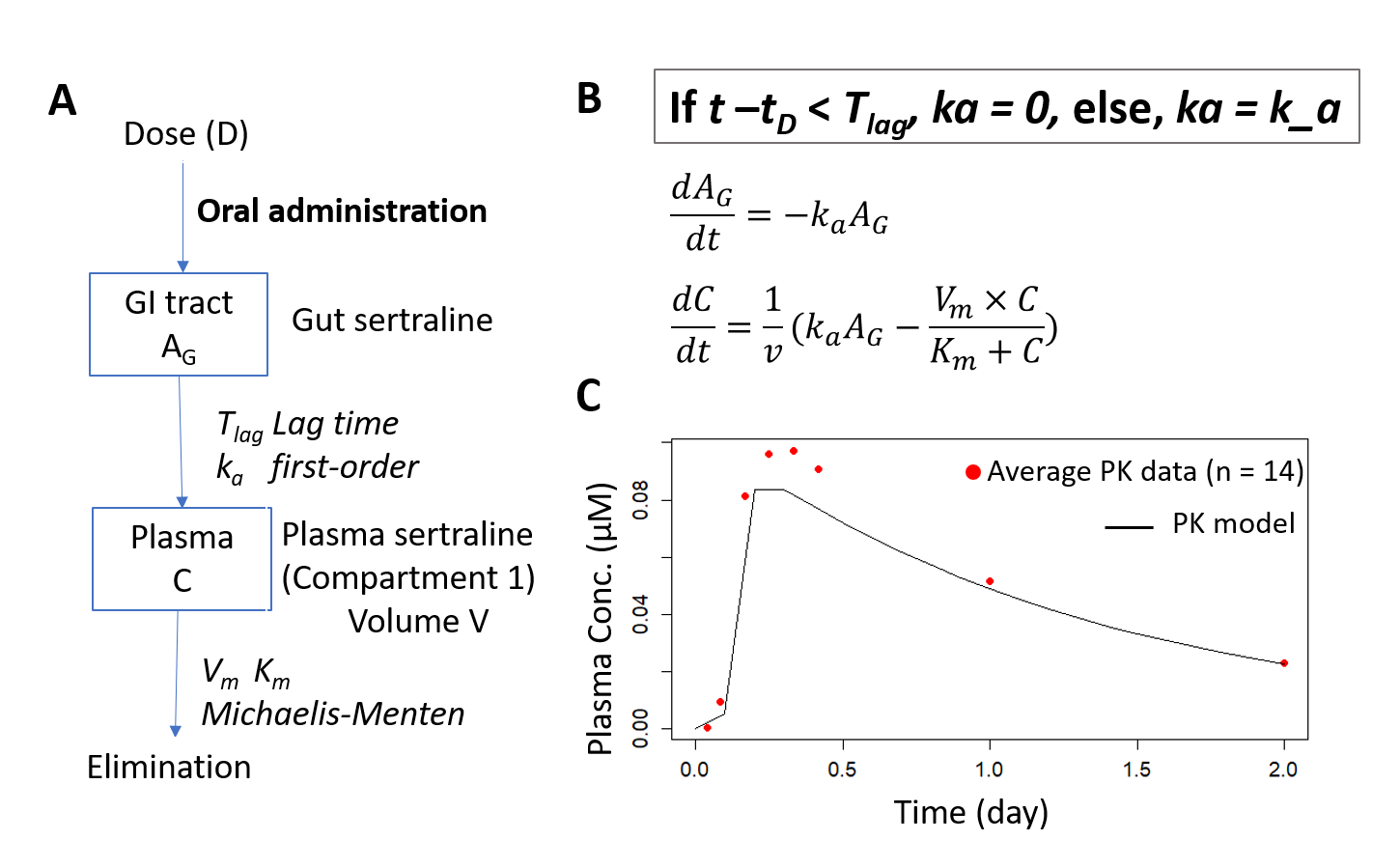

Supplement: Supplementary file 1 [file microorganisms-09-00566-s001.zip › microorganisms-1123604-S/Supplementary Figures/SupFig2.tif]

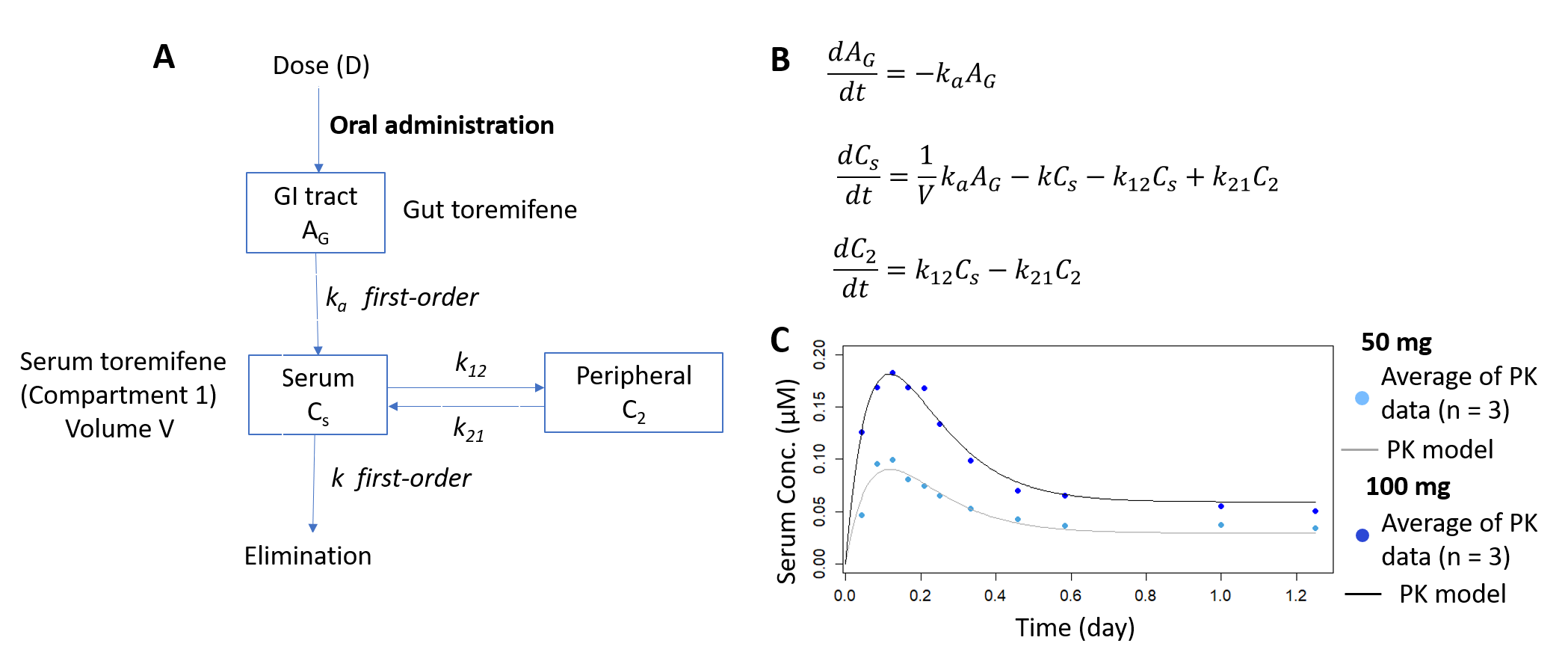

Supplement: Supplementary file 1 [file microorganisms-09-00566-s001.zip › microorganisms-1123604-S/Supplementary Figures/SupFig3.v1.tif]

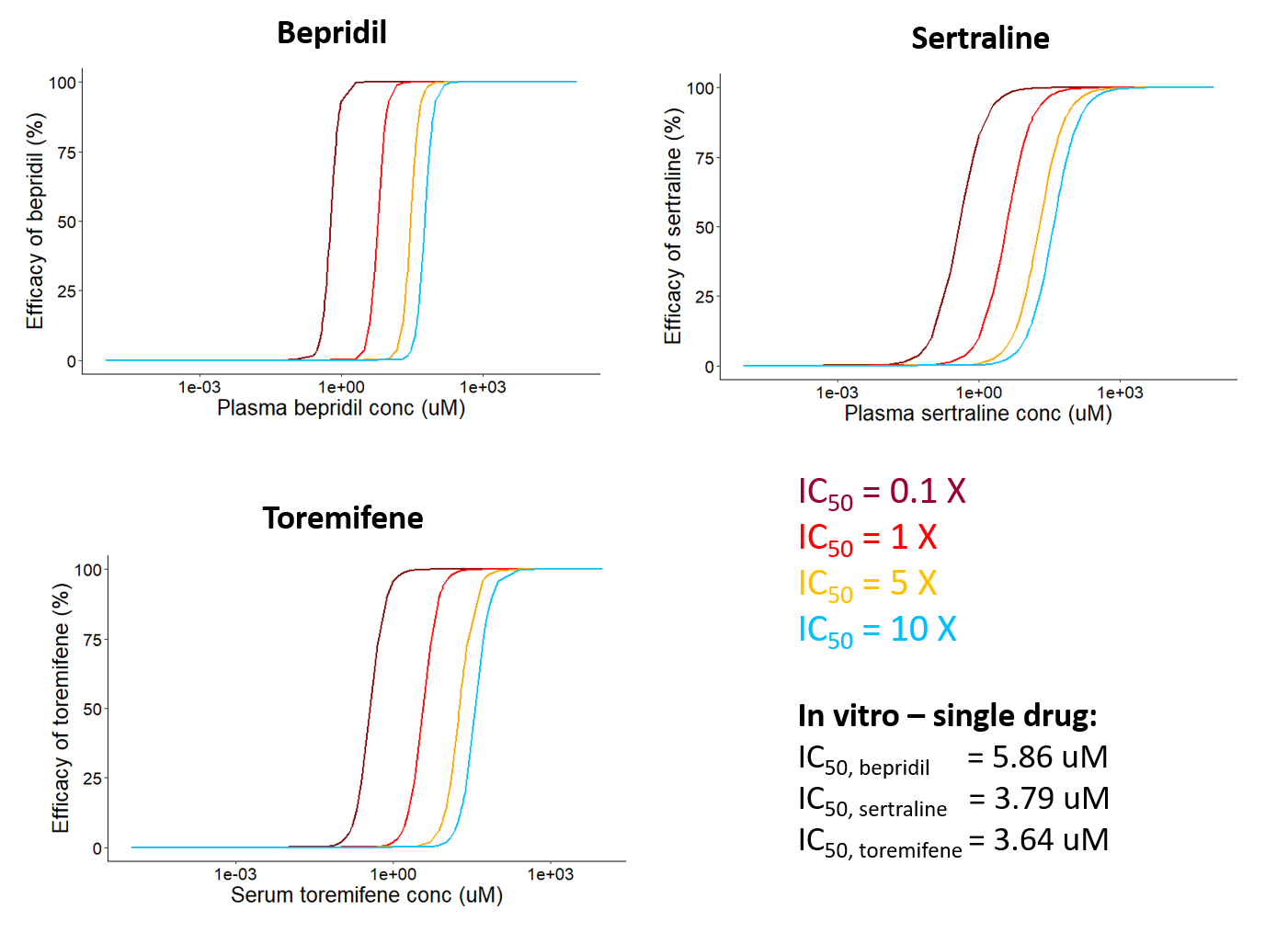

Supplement: Supplementary file 1 [file microorganisms-09-00566-s001.zip › microorganisms-1123604-S/Supplementary Figures/SupFig4.tif]

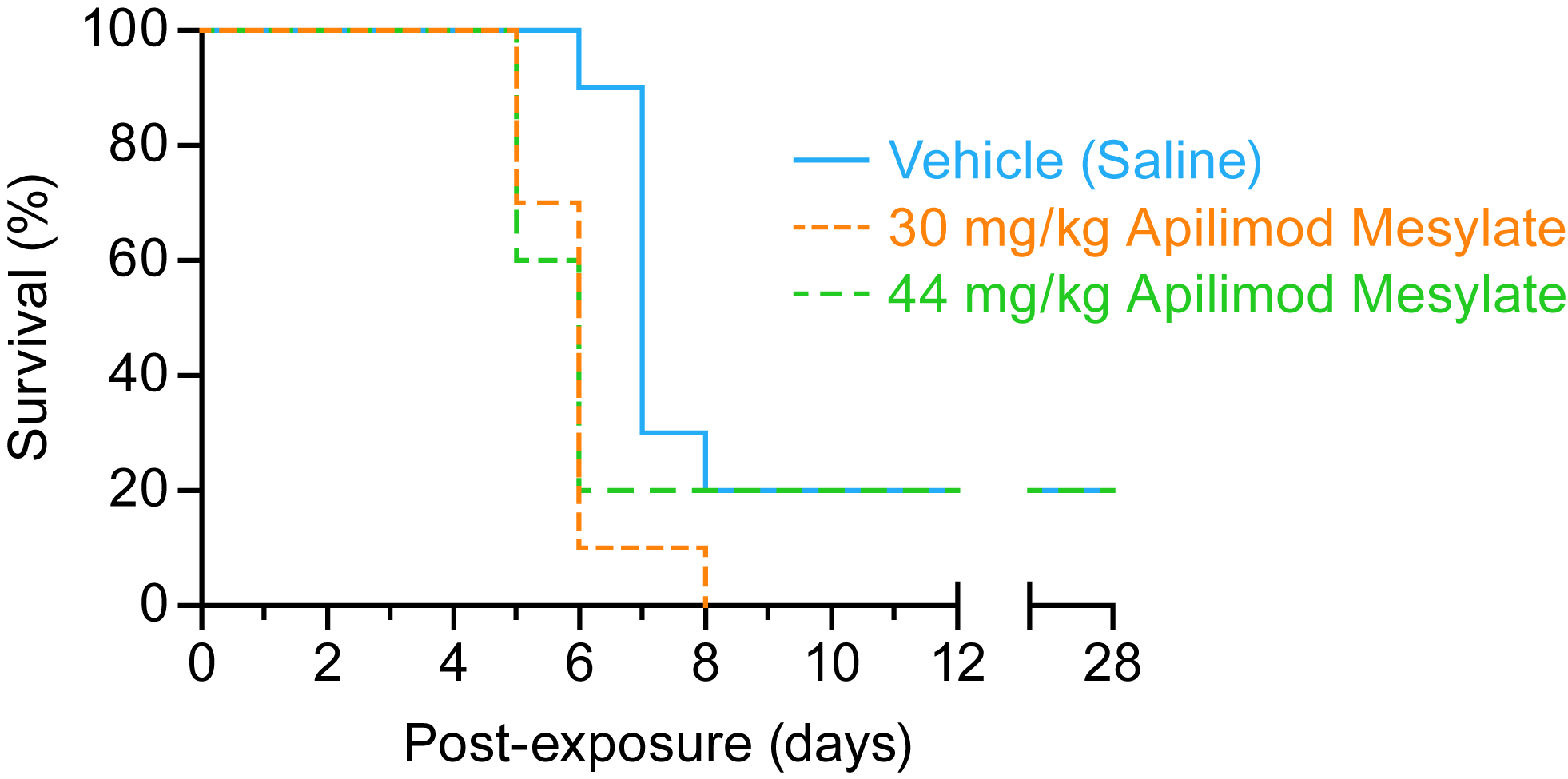

Supplement: Supplementary file 1 [file microorganisms-09-00566-s001.zip › microorganisms-1123604-S/Supplementary Figures/SupFig5.tif]
